# Sustainable and rapid preparation of nanosized Fe/Ni-pentlandite particles by mechanochemistry†

**DOI:** 10.1039/d0sc04525j

**Published:** 2020-11-05

**Authors:** David Tetzlaff, Kevinjeorjios Pellumbi, Daniel M. Baier, Lucas Hoof, Harikumar Shastry Barkur, Mathias Smialkowski, Hatem M. A. Amin, Sven Grätz, Daniel Siegmund, Lars Borchardt, Ulf-Peter Apfel

**Affiliations:** Fraunhofer UMSICHT Osterfelder Straße 3 DE-46047 Oberhausen Germany Ulf-peter.apfel@umsicht.fraunhofer.de; Ruhr University Bochum, Inorganic Chemistry I Universitätsstraße 150 DE-44780 Bochum Germany ulf.apfel@rub.de; Cairo University, Chemistry Department 1 Gamaa St. EG-12613 Giza Egypt

## Abstract

In recent years, metal-rich sulfides of the pentlandite type (M_9_S_8_) have attracted considerable attention for energy storage applications. However, common synthetic routes towards pentlandites either involve energy intensive high temperature procedures or solvothermal methods with specialized precursors and non-sustainable organic solvents. Herein, we demonstrate that ball milling is a simple and efficient method to synthesize nanosized bimetallic pentlandite particles (Fe_4.5_Ni_4.5_S_8_, Pn) with an average size of *ca.* 250 nm in a single synthetic step from elemental- or sulfidic mixtures. We herein highlight the effects of the milling ball quantity, precursor types and milling time on the product quality. Along this line, Raman spectroscopy as well as temperature/pressure monitoring during the milling processes provide valuable insights into mechanistic differences between the mechanochemical Pn-formation. By employing the obtained Pn-nanosized particles as cathodic electrocatalysts for water splitting in a zero-gap PEM electrolyzer we provide a comprehensive path for a potential sustainable future process involving non-noble metal catalysts.

## Abstract 2

A sustainable and rapid mechanochemical method for the preparation of bimetallic nanosized pentlandite particles as cathode material is developed and tested within zero-gap PEM cells.

## Graphical abstract

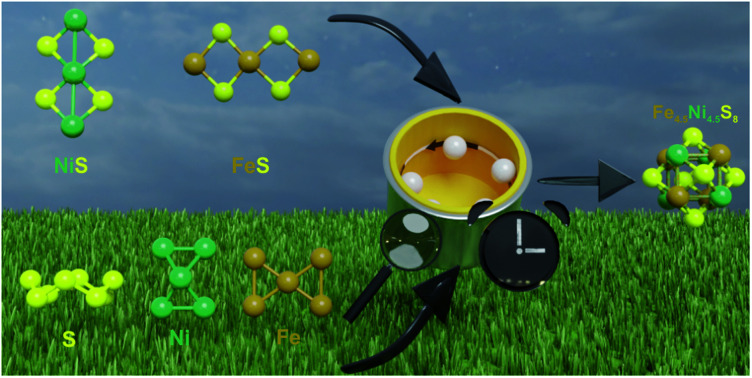

## Introduction

Metal-rich transition metal chalcogenides, in particular of the pentlandite type [M_9_S_8_, *e.g.* M = Fe, Co, Ni], recently emerged as promising materials for catalytic purposes^1,2^ (*e.g.* for CO_2_ reduction^3,4^ and water splitting^5–12^), energy storage applications^13,14^ as well as magnetic devices.^15^ This widespread interest can be attributed to the chemically robust nature of pentlandites, its pseudo-metallic conductivity as well as their large flexibility of stoichiometric compositions.^16^

Despite of the promising properties of pentlandite-type materials, their widespread and large-scale application is severely restricted by the currently employed synthetic strategies for their generation. Commonly, pentlandites are synthesized by high-temperature (*ca.* 1000 °C) solid-state reactions under exclusion of air in sealed containers;^6,7,17,18^ a highly inefficient method, especially in terms of time, energy input and waste production. Alternative approaches include hydro/solvothermal coprecipitation methods^13,19^ usually followed by an additional annealing^9–11^/sulfidation^14^ step with H_2_S^1^ or gaseous sulfur.^5^ Although these methods potentially yield well-defined nanoparticles, they suffer from an unreliable control of stoichiometry, require specialized precursors or unsustainable organic solvents.

In recent years, mechanochemistry emerged as a promising approach to overcome the aforementioned issues typically associated with the classical synthesis routes. In general, mechanochemical reactions proceed through the transfer of kinetic energy from impacting balls to powdery particles and allow for a rapid and precise synthesis of nanosized particles (NSPs) in a single step.^20,21^ The attractiveness for such an approach for industrial applications is further strenghtened by two major advantages:^22,23^ First, large scale tools for mechanochemistry such as extruders and vibratory mills are well-established and allow for a reliable scaleability of the process. Second, sustainable reaction conditions can be assured by avoiding complementary additives or organic solvents. Moreover, mechanochemical methods can proceed *via* reaction pathways unaccesible by classical synthetic methods.^24–26^

Previously, ball milling of complex Pn-compositions was only applied by a top-down approach to achieve sufficiently small particles and can be regarded as unoptimized due the necessity of two consecutive reaction steps and production of particles with sizes of 1–10 μm.^27^ Contrary, it has been shown that monometallic Co_9_S_8_ with sizes around 500 nm can be directly produced by a bottom-up approach from the respective elements or sulfides using mechanochemistry.^28,29^ Dutkova *et al.* improved the mechanochemical Co_9_S_8_ synthesis demonstrating that the reaction time can be decreased to a minimum of 90 min.^15^ However, direct mechanochemical synthesis of multimetallic complex pentlandites has not yet been realized and hence a potential controllability of a precise stoichiometric composition for (M_1_M_2_)_9_S_8_-type materials remains inconclusive.

We herein present mechanochemical protocols for the stoichiometric precise synthesis of bimetallic pentlandite NSPs starting from elemental and sulfidic reaction mixtures. The synthesized materials are thoroughly characterized and detailed mechanistic investigations are conducted to identify optimal reaction conditions and to further advance the mechanistic understanding of the mechanochemical syntheses. Ultimately, the synthesized Pn-NSPs are put to a practical test by employing the material as cathodic catalyst in a zero-gap PEM-type cell for the electrocatalytic hydrogen evolution reaction (HER) utilizing a catalyst coated substrate (CCS) approach.

## Results and discussion

The mechanochemical syntheses were performed in a planetary ball mill using 10 mm ZrO_2_ milling balls (*m* = 2.99 g) in 20 mL ZrO_2_ milling vessels. To prevent oxidation of the samples, the reaction vessels were filled with the reactant mixtures under an inert atmosphere of Ar. As reactants, a mixture of the pure elements (Fe, Ni, S) or their corresponding metal sulfides, which are recognized as notable intermediates in the traditional synthesis of Pn (FeS, NiS), was used.^30^

To achieve an optimal reaction progression with complete conversion, we opted for a near maximum rotation speed of 800 rpm for all reactions. Based on the results by Dutkova *et al.*, milling investigations were performed in time steps of 15 min until full conversion was observed. In addition, we investigated the effect of the ball-to-powder ratio by varying the number of added zirconia milling balls (six, eight or ten balls per vessel). We will refer to samples starting from elemental mixtures as E_*X*_-*Y* and the corresponding sulfides will be denoted as S_*X*_-*Y*, where *X* is the number of added milling balls to the milling vessel and *Y* is the milling time.

### Reaction progression and product analysis

**Fig. 1:**
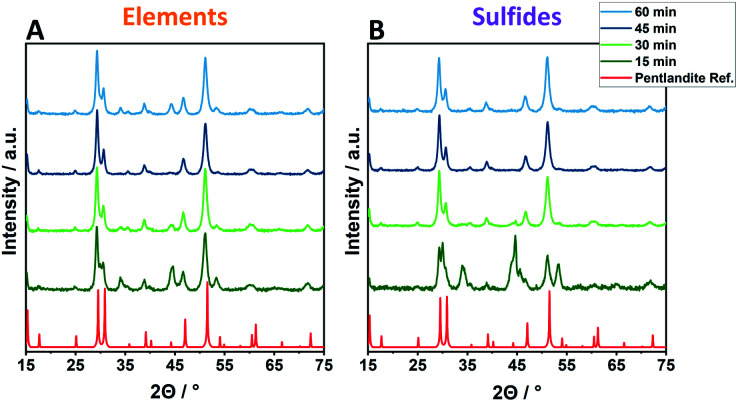
Exemplified powder X-ray diffractograms of Pn-materials synthesized at different time scales (15–60 min) using 8 × 10 mm ZrO_2_ balls and starting from an elemental reaction mixture (A, E_8_-*Y*) or the respective metal sulfides (B, S_8_-*Y*).

Employing reactant mixtures consisting of the pure elements, Fe, Ni and S, mixed in the proper stoichiometric amounts (E_*X*_-*Y*, see Experimental section for details), a strong dependence on the milling time and the ball-to-powder ratio is observed. Powder X-ray diffractograms (PXRD) of samples employing 8 balls are given exemplarily in Fig. 1 (complete PXRD data of all samples are given in the ESI†). After 15 min of milling time (E_*X*_-15), no phase-pure pentlandite can be observed in the powder diffractograms (Fig. 1A, S1 & S2A†). While E_6_-15 shows only reflexes belonging to elemental iron and nickel, an increased ball-to-powder ratio in E_8_-15 and E_10_-15 leads to the composite phases consisting of mixtures of pentlandite and FeNiS_2_ (Fig. 1A & S1C, S2A†). The stepwise increase of milling time leads to higher yields of pentlandite across all investigated E_*X*_-samples. In detail, E_6_-samples require a minimum of 30 min for Pn formation, with a Pn-yield of 62.1%, as determined *via* Rietveld analysis (Fig. S3, Table S1†). The Pn-phase becomes progressively more dominant as the milling time increases and reaches a Pn-yield of 87.5% after 60 min of milling accompanied by other sulfidic impurities. Notably, using eight and ten milling balls the pentlandite phase already becomes dominant after 30 minutes of milling with Pn-yields of 86.1 and 85.7%, respectively (Fig. 1A & S1C, Table. S1†). The diffractograms of E_8_-45 and E_10_-45 exclusively show the desired pentlandite phase. As anticipated, increasing the number of collisions by transitioning from six to eight balls leads to a higher Pn-phase generation at comparable milling times. This effect is of minor significance when transitioning from eight to ten balls thereby potentially reflecting a somewhat decreased average energetic input per collision event due to a smaller mean free path of the milling balls. It is to be expected, that this phenomenon will dominate with even more milling balls ultimately hampering an efficient pentlandite formation. Thus, the addition of more than 10 milling balls was not performed in this study.

Samples synthesized from the respective metal-sulfides (FeS and NiS) instead of pure elements show overall trends comparable to elemental reactants. However, an altered time-dependent product formation is observed (Fig. 1B & S1D–F, Table S2†). Remarkably, almost quantitative conversion using sulfidic reactants can already be achieved within 30 minutes as evidenced by the samples S_8_-30 (90.7%) and S_10_-30 (99.7%), hereby reducing the required reaction time drastically as compared to elemental mixtures (45 minutes for E_8_ or E_10_).

To confirm the purity of the synthesized Pn-type materials, the samples were subjected to differential scanning calorimetry (DSC) analysis. As expected, the synthesized materials display a characteristic pentlandite phase transitions at ∼613 °C (transition from a low to a high-temperature Pn form) and at ∼863 °C (phase breakdown) (Fig. S4, Tables S3 & S4†).^31^ All synthesized materials, except of E_6_-15 (Fig. S4A†), show similar phase transitions regardless of the phase purity determined *via* powder XRD. This behavior is attributed to a thermal completion of Pn formation during DSC-analysis facilitated by an increased surface area and structural defects induced by ball milling. In addition, sample E_6_-15 shows a strong exothermal signal around 200 °C, which can be attributed to unreacted sulfur within the sample (Fig. S4†). Thus, DSC analysis complements the PXRD data by revealing unreacted elemental impurities in the synthesized samples and confirms reaction completeness for samples with longer milling times.

**Fig. 2:**
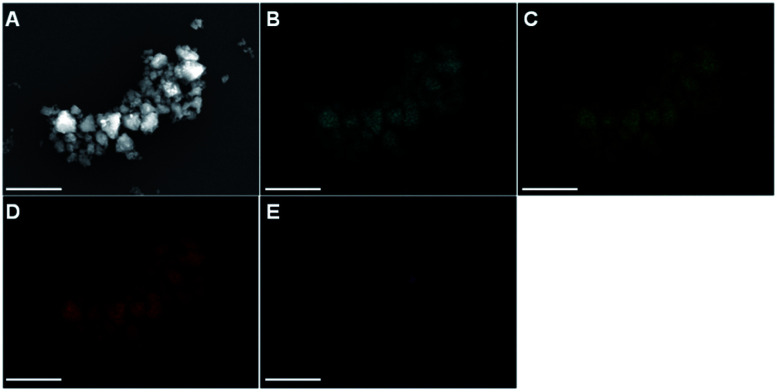
(A) SEM image displaying the particles sizes and morphologies of E_8_-45. Further, EDX mappings reveal the presence of the elements (B) Fe (blue), (C) Ni (green), (D) sulfur (red) and (E) zirconium (violet). The scale bar is 2.5 μm.

Scanning electron microscopy (SEM) characterization of the obtained materials reveals comparable particle morphologies and sizes for all tested samples (Fig. S6 & S7†). As evidenced by the representative sample E_8_-45, irregularly shaped particles with a non-uniform size-distribution between 0.1–1 μm (Fig. 2A) are formed. Furthermore, increasing the ball-to-powder ratio as well as milling time results in smaller and more regular particles (Fig. S6 & S7†). A more detailed picture of the size distribution of the obtained pentlandite particles is given by the disc centrifuge sedimentation (DCS) analysis (Fig. S8†) and confirms the formation of nanosized particles below 400 nm (Tables S5 & S6†). The majority of samples even yield Pn-particles smaller than 250 nm at a maximum of 60 minutes milling time.

### Quantification of mechanical abrasion impurities

**Fig. 3:**
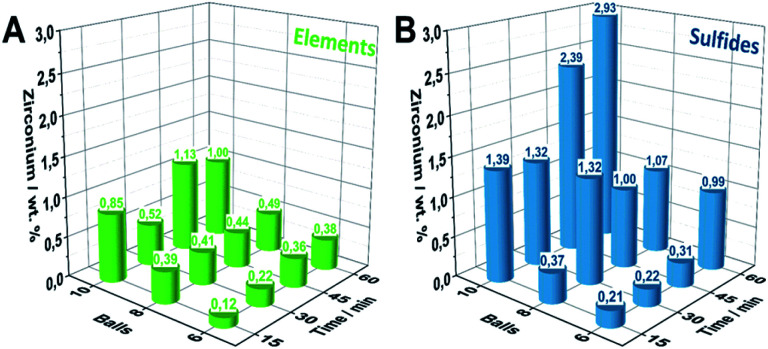
Quantification of Zr-abrasion impurities *via* ICP-OES of the synthesized Pn-samples by employing (A) elemental reaction mixtures. (B) Sulfidic reaction mixtures.

Extensive ball milling can lead to mechanical abrasion of the milling container and balls causing considerable contamination of the reaction product and potentially influences the material performance.^32^ As this must not be neglected in mechanochemical studies, we herein employed inductively coupled plasma optical emission spectroscopy (ICP-OES) measurements for Zr-quantification for all samples. Within the E_6_-*Y* series, increasing milling times lead to a gradually increasing accumulation of Zr from 0.12 wt% to 0.38 wt% (Fig. 3A). A similar behavior is also observed for samples series E_8_-*Y* and E_10_-*Y* reaching values of 0.49 wt% and 1.00 wt% of Zr, respectively. Notably, employing the sulfidic reaction mixture as the starting composition leads to a significantly higher Zr accumulation compared to pure elemental reactants. However, employing sulfidic reaction mixtures, the S_6_-*Y* series likewise shows a gradual Zr-increase (Fig. 3B). Starting from 0.2 wt% for S_6_-30 the Zr-content increases to about 0.99 wt% for S_6_-60. For samples beyond S_8_-30 (1.32 wt%), the amount of Zr decreases to 1 wt% for S_8_-45 and S_8_-60. However, milling sulfidic reactants with 10 milling balls yields the highest Zr-impurities starting of up to 2.93 wt% for S_10_-60.

The difference in the Zr-content of the S- and E-series can be attributed to the higher density and hardness of the employed sulfidic reaction mixtures compared to its elemental reaction mixtures.^33^ The ICP-OES results were further supported in trend and magnitude by particle induced X-ray emission (PIXE) measurements serving as an independent method for the Zr-quantification (Fig. S9†).

Apart from the Zr-quantification, it is important to understand how the abrased impurities are incorporated in the synthesized pentlandite materials. Fig. 2B–E displays an energy dispersive X-ray spectroscopy (EDX) mapping analysis complementarily to the previously discussed SEM image. It is evident that the signals from the elements Fe, Ni and S appear at the exact position of the expected Pn-particles in the SEM image. Furthermore, it is important to note that the elements are equally distributed among all particles which confirms precise control over the desired product stoichiometry (here a 1 : 1 ratio of Fe and Ni). In contrast, Zr is only present as a single spot in the EDX image, thereby suggesting that the impurities exist as isolated particles alongside the pure Pn particles. Furthermore, this observation shows that Zr is not integrated in the crystal lattice of pentlandite and is consequently not affecting the materials intrinsic properties (Fig. S10–S15†).

Since the rate of zirconia abrasion tends to increase with milling time and number of milling balls, the results underline the necessity for careful trade-off between energy input/reaction time as well as choice of starting materials to minimize the amount of abrased milling material. While reactants based on elemental mixtures lead to considerably less Zr-impurities as compared to sulfidic reactants at comparable milling times, the milling time to achieve complete conversion might be considerably longer.

### Mechanistic insights

**Fig. 4:**
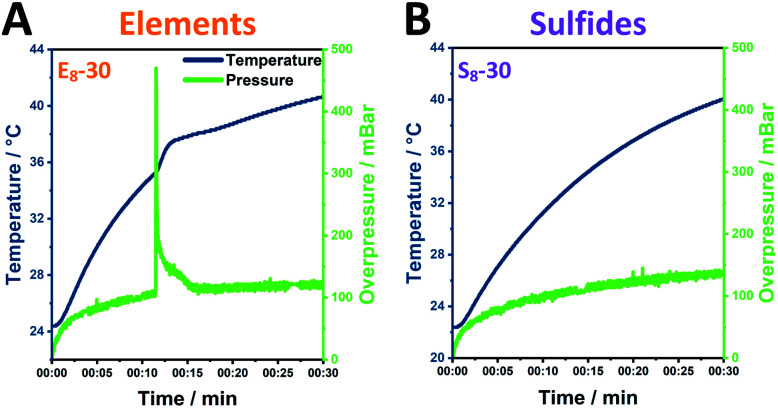
Pressure- and temperature measurements during Pn synthesis employing (A) eight 10 mm ZrO_2_ balls added to an elemental reaction mixture; (B) eight 10 mm ZrO_2_ balls added to a sulfidic reaction mixture.

In order to understand the processes in the milling vessel, we monitored the temperature and pressure changes during the mechanochemical reaction for 30 minutes of milling. The investigated element samples show the reaction profile of a mechanochemically induced self-sustaining reaction (MSR)^34–39^ (Fig. 4A, S16A–C†). This profile is often observed in mechanochemical reactions and especially sulfide materials are prone to this behavior.^38^ Initially, the pressure and temperature rise slowly. In this mechanical induction phase, both the surface area and the number of structural defects in the grinding material increase, which enhances its reactivity. Once enough energy has been accumulated to start the highly exothermic reaction, the released heat is sufficient to maintain the reaction progress. This combustion-like event is associated not only with a rise in temperature but also with a sharp rise in pressure.

The ignition time at which the pressure starts to rapidly increase significantly changes as the ball-to-powder ratio is varied and decreases from approximately 15 min (E_6_) to 12 min (E_8_) and ultimately to 8 min (E_10_). Similarly, the heating slope at the ignition point is increased with increasing number of milling balls. This behavior can be attributed to the higher number of collisions induced by the increasing number of milling balls in the reaction vessel.^38^

In contrast, sulfide mixtures do not demonstrate this MSR behavior (Fig. 4B, S16D–F†). The relative pressure herein gradually increases alongside the temperature of the milling vessel with both samples, S_8_-30 and S_10_-30, reaching an overpressure of 140 mbar and 100 mbar (S_6_-30).

We attribute the emergence of this MSR to the reactive nature of the two metals with sulfur. Therefore, a possible explanation of the underlying mechanism is that after the ignition time the reaction proceeds through a series of mixed sulfide phases before reaching the thermodynamically stable pentlandite phase.^40^ To further test this theory, the reaction was stopped closely before and after the rapid pressure increase employing 8 milling balls and subsequently examined the reaction mixture. Powder diffractograms revealed that the reflexes at 35° and 45° which correspond to (Fe,Ni)S_2_ phases significantly decrease in intensity, whereas the reflex at 46° corresponding to NiS is not detected after the start of the MSR reaction (Fig. S17†). We hypothesize that the shorter reaction times at which pentlandite can be obtained through sulfides is the result of already employing intermediates that would otherwise first need to be generated from the mechanochemical reaction of Fe, Ni and S.

**Fig. 5:**
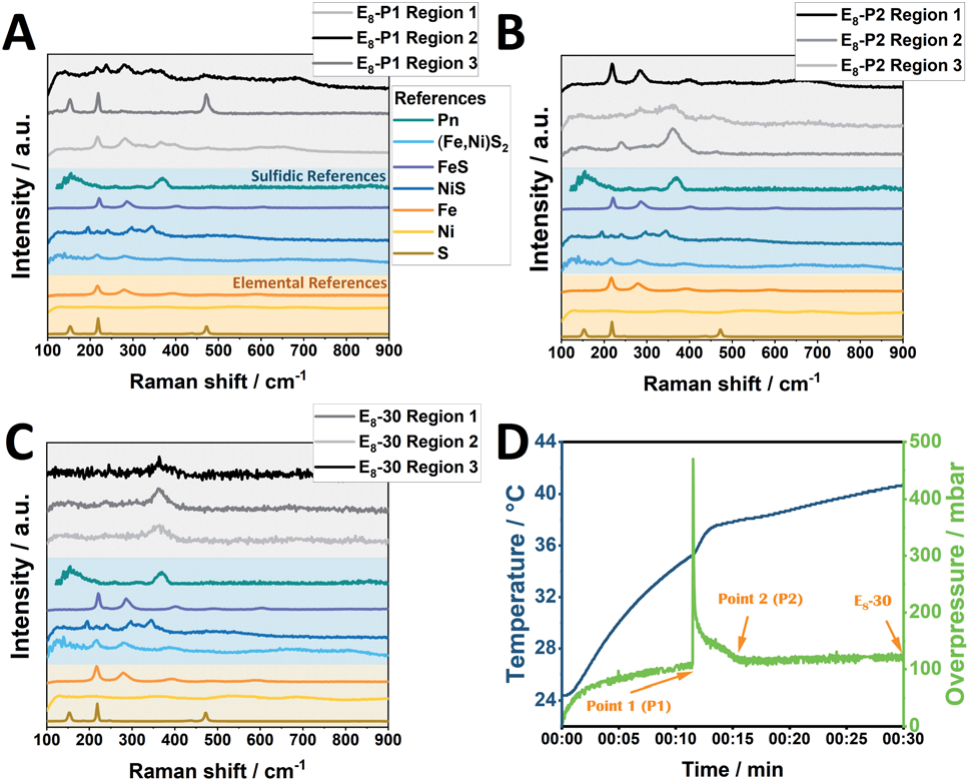
Raman spectra of the obtained E_8_-*Y* samples at three different time points of the mechanochemical reaction: closely before the initiation of the MSR reaction (A), closely after (B) and after 30 min of milling (C). The respective points are also shown for clarity in (D).

Notably, Raman spectra collected after the completion of the pressure peak at three different regions (measured at three independent spots of the powder sample, denoted as region 1–3) likewise demonstrate the generation of Pn phases along with FeS and NiS demonstrating that the reaction is not fully completed in the milling vessel, as demonstrated by the PXRD data of E_8_-15 (Fig. 5). For comparison, in case of E_8_-30 only signals belonging to Pn are observed. Regarding the sulfidic counterpart, terminating the mechanochemical reaction at the half-way point yielded a sample consisting mainly of FeS, NiS and (Fe,Ni)S_2_ according to our Raman analysis with peaks corresponding to the Pn phase not being detected (Fig. S18†).

Comparing the elemental and sulfidic mixtures, it is worth mentioning that potential hazards from sharp increasing pressures are avoided by employing sulfidic reactants. However, this advantage needs to be balanced against the Zr-abrasion, which is considerably lower for elemental reactants.

### Electrochemistry

In recent years, pentlandite-based materials have demonstrated significant potential as electrocatalysts for the hydrogen evolution reaction (HER). Previous investigations were mainly performed with rod-type electrodes consisting of the pure material without additives.^6,7,18^ Applying these materials, prepared from a cheap and sustainable method, in a PEM-electrolyzer now constitutes a vital step towards their potential future commercial applications. For our experiments, we employed an in-house build electrochemical PEM-cell (Fig. S19†) and selected the Pn-catalyst E_8_-45 which showed full conversion of starting materials to form the desired product phase at the lowest milling time while keeping the amount of Zr-impurities at a minimum compared to similar phase-pure samples.

Catalyst inks were prepared by mixing the materials with carbon black, Nafion as ionomer binder (16.7 wt%) and were air-brushed onto a commercial carbon cloth gas diffusion layer equipped with a microporous layer (MPL). For the anode (OER), catalyst coated membranes were prepared by air-brushing Ir-black mixed with Nafion onto a Nafion HP (20 μm) membrane.

**Fig. 6:**
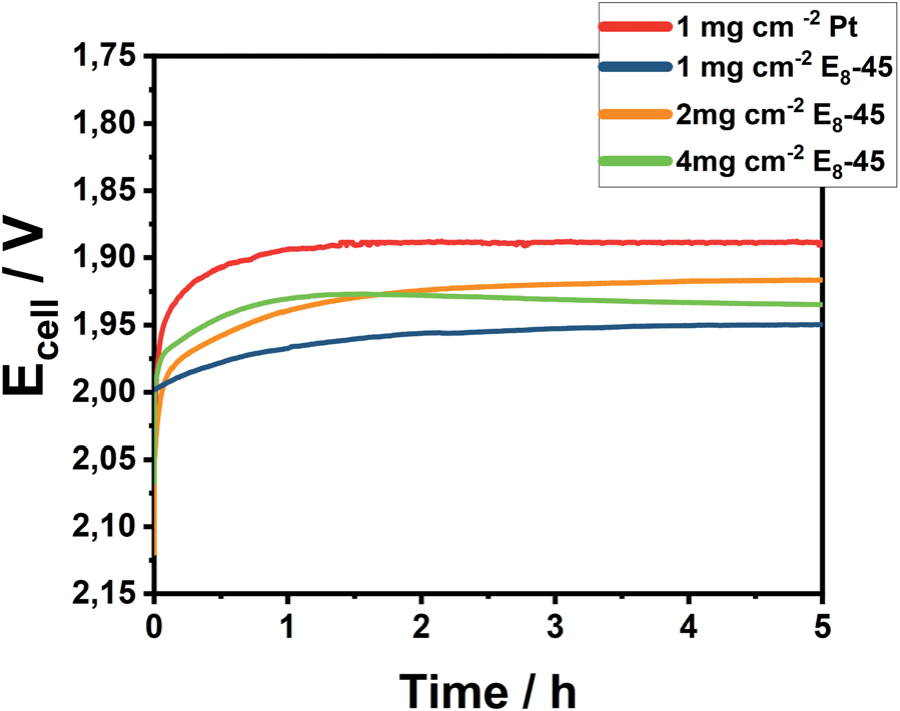
Chronopotentiometry of Pt/C and E_8_-45/C at a catalytic loading of 1–4 mg cm^−2^ at 80 °C for 5 h at an applied current of 1 A cm^−2^.

The electrocatalytic investigations were performed at 80 °C using a flow of Milli-Q water supplying both cell compartments. At a catalyst loading of 1 mg cm^−2^, an appreciable electrocatalytic activity was observed (Fig. 6 and S20†) reaching the target current density of 1 A cm^−2^ at a cell potential of 1.95 V. After an initial activation phase, the cell potential remained constant during the 5 h electrolytic experiment. Further increasing the catalytic loading closer to those previously reported on metal sulfides^41–44^ (3–4 mg cm^−2^) results in improved potentials of 1.91 V and 1.93 V for 2 mg cm^−2^ and 4 mg cm^−2^ at the end of electrolysis, respectively. The lower performance of electrodes with 4 mg cm^−2^ might stem from an unoptimized mass transport within the thick electrode. Notably, the Pn-catalysts demonstrate a lower cell voltage as compared to similar Fe/Ni-sulfidic catalysts (Table S7†).^44^

In addition, we examined the effect of the preparation method of the catalyst by comparing the activity of a top-down generated pentlandite, *i.e.* a thermally synthesized and mechanochemically milled pentlandite, to E_8_-45. The top-down pentlandite reaches the target current density at a slightly higher cell potential (Δ*E* = 30 mV) compared to the mechanochemically generated material (Fig. S21†) confirming a similar catalytic activity. The observed minor differences are attributed to differences in the catalysts particle sizes as a result of the preparation method.

Transitioning beyond rod electrodes, Pn was clearly shown to possess promising properties for its application as HER catalyst in a PEM setup. Importantly, the herein presented synthesis provides a facile and scalable method towards a commercial product. We are confident that further optimization of the cell configuration as well as synthetic parameters will lead to dramatic improvements of the catalytic efficiency of mixed-metal pentlandites in PEM electrolysis ultimately approaching the activity of Pt-based catalysts.

## Conclusion

In summary, we herein present the rapid and solvent-free preparation of phase-pure pentlandites by a green and cost-effective mechanochemical approach using a ball mill. To the best of our knowledge, this is the first report utilizing mechanochemistry for synthesizing bimetallic sulfides of the Pn type. Compared to traditional synthesis for these materials, employing milling enables a more precise control of the materials' composition and can easily be adopted for altered stoichiometric formulations. At the same time completely sustainable reaction conditions are assured by avoiding organic solvents as well as the need for long-term high-energy input.

The reaction conditions were optimized by the systematic variation of decisive parameters such as ball-to-powder ratio, milling time as well as reactants, which altogether influence the products' phase purity. Importantly, by quantification of Zr-impurities caused by mechanical abrasion we raise awareness of a potential parasitic effect often neglected in mechanochemical studies.

Going further, we strengthen the understanding of mechanochemical reaction progressions by providing detailed Raman-measurements as well as continuous temperature/pressure monitoring which shed light on the Pn formation process. Pn synthesis from elemental mixtures likely proceeds through the formation of mixed metal sulfides through a MSR-type mechanism. The accompanying rise in pressure and temperature can be avoided by using sulfide-mixtures as reactants at the cost of higher Zr-abrasion.

Finally, to demonstrate the applicability of the synthesized Pn-material in electrocatalytic applications, the obtained product E_8_-45 was for the first time employed as a cathodic hydrogen evolution catalyst in a zero-gap PEM cell at 1 A cm^−2^ for an extended period of time. The results clearly demonstrate the potential for the as obtained catalyst particles to compete with traditional Pt-based catalysts systems for the efficient sustainable formation of dihydrogen by water splitting in the near future. Taken together we believe that this work outlines a clear environmentally friendly and scalable strategy for obtaining non-noble metal materials with widespread usage in industrial applications.

## Experimental

### Materials

Elemental iron, nickel, sulfur, iron(ii) sulfide, nickel(ii) sulfide, Pt/C (40%), and Ir-black were purchased from commercial vendors and used without further purification.

### Synthesis

Synthesis of pentlandite (Fe_4.5_Ni_4.5_S_8_) was performed using a Fritsch Pulverisette 7 premium line planetary ball mill with ZrO_2_ milling containers (*V* = 20 mL) and ZrO_2_ milling balls (*d* = 10 mm, *m* = 2.99 g). The reaction mixtures (*m* = 1 g) were composed of either stoichiometric amounts of the elements Fe (*m* = 0.326 g), Ni (*m* = 0.342 g) and S (*m* = 0.332 g) or the sulfides FeS (*m* = 0.456 g), NiS (*m* = 0.470 g) with addition of the necessary stoichiometric amounts of Fe (*m* = 0.036 g) and Ni (*m* = 0.038 g). An inert argon (Ar) atmosphere inside the milling containers was established by preparing the mixtures inside a glovebox. The mechanochemical reactions were performed at a constant rotation speed of 800 rpm and a variable amount of milling balls (6, 8 and 10; ball-to-powder ratio 17.94 : 1, 23.98 : 1 and 29.90 : 1) and milling times (15, 30, 45 and 60 min).

For the temperature- and pressure monitoring measurements, a commercial EASY GTM gas pressure and temperature measuring system was used. Prior to investigation an inert argon (Ar) atmosphere inside the milling containers was established by preparing the mixtures inside a glovebox. Milling was performed for 30 min at 800 rpm. The special lid and transmitter in combination with the MillControl software enabled visualizing temperature and pressure during the grinding process.

### Characterization

Powder X-ray diffraction (PXRD) was performed on an X'Pert Powder X-ray diffractometer from Malvern Panalytical using a Cu radiation source (*λ* = 1.5406 Å) at 45 kV and 20 mA at a step size of 0.02°. Rietveld analysis was performed using the HighScore Plus (Malvern Panalytical) software.

Differential scanning calorimetry (DSC) data were recorded utilizing a Netzsch STA 449 F3. Approximately 50 mg of sample substance were placed in a corundum crucible and heated from 27 °C up to 1050 °C at a heating rate of 10 K min^−1^. Nitrogen (N_2_) was applied as purging gas at a flow rate of 50 mL min^−1^. The balance was protected by a nitrogen purge of 20 mL min^−1^. As a reference an empty crucible plus lid was used. Temperature and device sensitivity were calibrated against high purity Ag, Au, In and Zn standards.

Inductively coupled plasma atomic emission spectroscopy (ICP-OES) was performed on an ARCOS II MV (SOP/EOP) (SPECTRO Analytical Instruments GmbH) with optional axial or radial plasma observation and elemental measurement in the wavelength range of 130–770 nm. Samples for elemental analysis were digested in a platinum crucible with the addition of 0.1 g of a lithium metaborate/tetraborate mixture at 1050 °C for 1 h. The melt is then taken up with HCl (diluted 1 : 6) for 1 h at 80 °C.

Scanning electron microscopy (SEM) was performed on a ZEISS Gemini2 Merlin HR-FESEM equipped with an OXFORD AZtecEnergy X-ray microanalysis system for energy dispersive X-ray spectroscopy (EDX). Prior to investigation, the samples were dispersed in 1 mL ethanol and ultra-sonicated for 30 min. Subsequently the samples were drop casted on a flat Si Wafer for analysis. The SEM images were recorded at an acceleration voltage of 4 kV while EDX mappings were performed from 0–20 kV.

Disc centrifuge sedimentation measurements were performed on a DC24000 UHR disc centrifuge instrument (CPS Instruments Inc., LA, USA). Prior to the measurement, a density gradient comprising 24 wt% and 8 wt% sucrose solutions was built within the disc and a disc rotation of 12 000 rpm was set. A sample suspension was prepared by dispersing the particles in ethanol, followed by sonication for 10 min. Each sample was calibrated against a poly(vinyl chloride) calibration standard with a diameter of 483 nm. 100 μL of the sample was injected into the disc. Two replicates were conducted for each sample. The CPS instrument software was used to generate the weight-based particle size distribution considering the particle density (4.9 g cm^−3^).

Raman spectroscopy was performed using a RENISHAW in VIA Qontor Raman microscope with 50× object (NA = 0.50, 8.2 mm free working distance). The spectra were collected with a laser wavelength of 532 nm and a laser power between 5 to 50% dependent on the sample. The exposure time was 0.5 s and 10 accumulations were collected.

Particle induced X-ray emission (PIXE) measurements were performed with a proton beam of 3 MeV and a beam intensity of 1–2 nA. The X-rays were detected with a silicon detector (Amptek) at an angle of 45° to the sample surface. Data analysis was performed through the use of the GUPIX software.

### Electrochemical measurements

For the electrocatalytic hydrogen evolution, catalyst inks were prepared by mixing 0.4 g of the catalyst under investigation, 0.6 g carbon Vulcan XC7R (Carbot) and 4.0 g of Nafion solution (5 wt%) (Sigma Aldrich). The ink was subsequently diluted by adding 7.88 g of Milli-Q water and 22.12 g ethanol. Inks containing Pt/C (40%) (Quintech) served as reference material and were prepared in a similar fashion. For the anode, 0.4 g of Ir-black were mixed with 4.0 g of Nafion solution (5 wt%) and diluted similarly. All catalytic inks were sonicated for 30 min and subsequently thoroughly mixed using an Ultra-Turrax T18D at 13.000 rpm for 1 min.

Conditioning of the Nafion HP membrane (Dupont) was performed by immersing the membrane in Milli-Q water.

For electrode preparation, a hand-held Iwata Eclipse with a compressed air carrier gas (∼100 mL min^−1^) was employed. For the anode preparation the Ir-ink was air-brushed at 80 °C onto a Nafion HP membrane (Dupont) until a loading of 2.5 mg cm^−2^ was reached. Cathodic electrodes were prepared in a similar fashion. The sulfidic catalyst was air-brushed on a carbon cloth equipped with MPL (CeTech) until the desired catalytic loading was achieved. Conditioning of the Nafion HP membrane (Dupont) was performed by immersing the membrane in Milli-Q water.

Hot-pressing of the catalyst coated membrane and cathodic electrode was performed separately at 110 °C for 30 seconds under a pressure of 0.1 kN cm^−2^ with the help of a Polystat 300S hot press. Electrochemical conditioning of the MEA was performed as described previously.^45^ Polarization curves were obtained by applying a series of current densities starting from 0.16 A cm^−2^ up to 1.12 A cm^−2^ in 160 mA cm^−2^ steps. Each current density was held for 5 min till a stable potential current density was obtained.

Membrane electrode assemblies (MEAs) were assembled in a single cell electrolyzer using electrodes of an area of 12.5 cm^−2^ with a torque of 5 Nm employing PTFE gaskets, stainless-steel flow fields and copper plates as anodic collector and cathodic collector feed respectively. During the electrochemical measurements Milli-Q water was pumped at 80 °C through both half-cells at a flow rate of 45 mL min^−1^. Polarization and chronopotentiometric curves were recorded on a Zahner Zennium potentiostat/galvanostat connected to a power potentiostat PP201.

## Conflicts of interest

There are no conflicts to declare.

## Supplementary Material

SC-011-D0SC04525J-s001
