# Supplementary material for: Sustainable and rapid preparation of nanosized Fe/Ni-pentlandite particles by mechanochemistry
Source: Chem Sci. 2020 Nov 5;11(47):12835–42. doi: 10.1039/d0sc04525j (PMC8163287; doi:10.1039/d0sc04525j)
Supplement: SC-011-D0SC04525J-s001 [file SC-011-D0SC04525J-s001.pdf]

Electronic Supplementary Material (ESI) for Chemical Science.
This journal is © The Royal Society of Chemistry 2020

Sustainable and Rapid Preparation of Nanosized Fe/Ni-Pentlandite Particles by Mechanochemistry

SUPPORTING INFORMATION

David Tetzlaff[a,b,†], Kevinjeorjios Pellumbi[b,†], Daniel M. Baier[b], Lucas Hoof[a], Harikumar Shastry Barkur[b], Mathias Smialkowski[b], Hatem M. A. Amin[b,c], Sven Grätz[b], Daniel Siegmund[a], Lars Borchardt[b] and Ulf-Peter Apfel*[a,b]

a) Fraunhofer UMSICHT, Osterfelder Straße 3, DE-46047 Oberhausen, Germany

b) Ruhr University Bochum, Inorganic Chemistry I, Universitätsstraße 150, DE-44780 Bochum, Germany

c) Cairo University, Chemistry Department, 1 Gamaa St., EG-12613 Giza, Egypt

† These authors contributed equally to this work.

Corresponding author.

**Email: ulf.apfel@rub.de or ulf.apfel@umsicht.fraunhofer.de*

**Email: lars.borchardt@rub.de*

Contents

Phase analysis of the synthesized Pn materials 4

Rietveld analysis of the synthesized Pn materials 5

Differential Scanning Calorimetry of the synthesized Pn materials 6

Phase transitions of the synthesized Pn materials from the elemental reactants according to Differential Scanning Calorimetry 7

Phase transitions of the synthesized Pn materials from the sulfidic reactants according to Differential Scanning Calorimetry 8

Differential Scanning Calorimetry analysis of an unreacted elemental reaction mixture 9

Scanning Electron Microscopy of the synthesized Pn materials from an elemental reaction mixture 10

Scanning Electron Microscopy of the synthesized Pn materials from a sulfidic reaction mixture 11

Disc centrifuge measurements of the synthesized materials 12

Particle size analysis of the synthesized Pn materials prepared through an elemental reaction mixture 13

Particle size analysis of the synthesized Pn materials prepared through a sulfidic reaction mixture 14

Non-noble metal HER electrocatalysts in a PEM electrolyzer reports in literature 15

Zr quantification through PIXE 16

EDX mapping analysis of the synthesized Pn materials prepared through an elemental reaction mixture using six ZrO_2 milling balls 17

EDX mapping analysis of the synthesized Pn materials prepared through an elemental reaction mixture using eight ZrO_2 milling balls 18

EDX mapping analysis of the synthesized Pn materials prepared through an elemental reaction mixture using ten ZrO_2 milling balls 19

EDX mapping analysis of the synthesized Pn materials prepared through a sulfidic reaction mixture using six ZrO_2 milling balls 20

EDX mapping analysis of the synthesized Pn materials prepared through a sulfidic reaction mixture using eight ZrO_2 milling balls 21

EDX mapping of the synthesized Pn materials prepared through a sulfidic reaction mixture using ten ZrO_2 milling balls 22

Gas and Temperature monitoring in the milling vessel starting from elemental and sulfidic reactant mixtures 23

PXRDs of the synthesized Pn materials generated through the Gas and Temperature monitoring experiments 24

Raman investigation of the S_8-Y samples 25

Electrochemical cell for PEM investigation 26

Electrochemical HER using the synthesized materials in a PEM electrolyzer 27
References 28

Phase analysis of the synthesized Pn materials

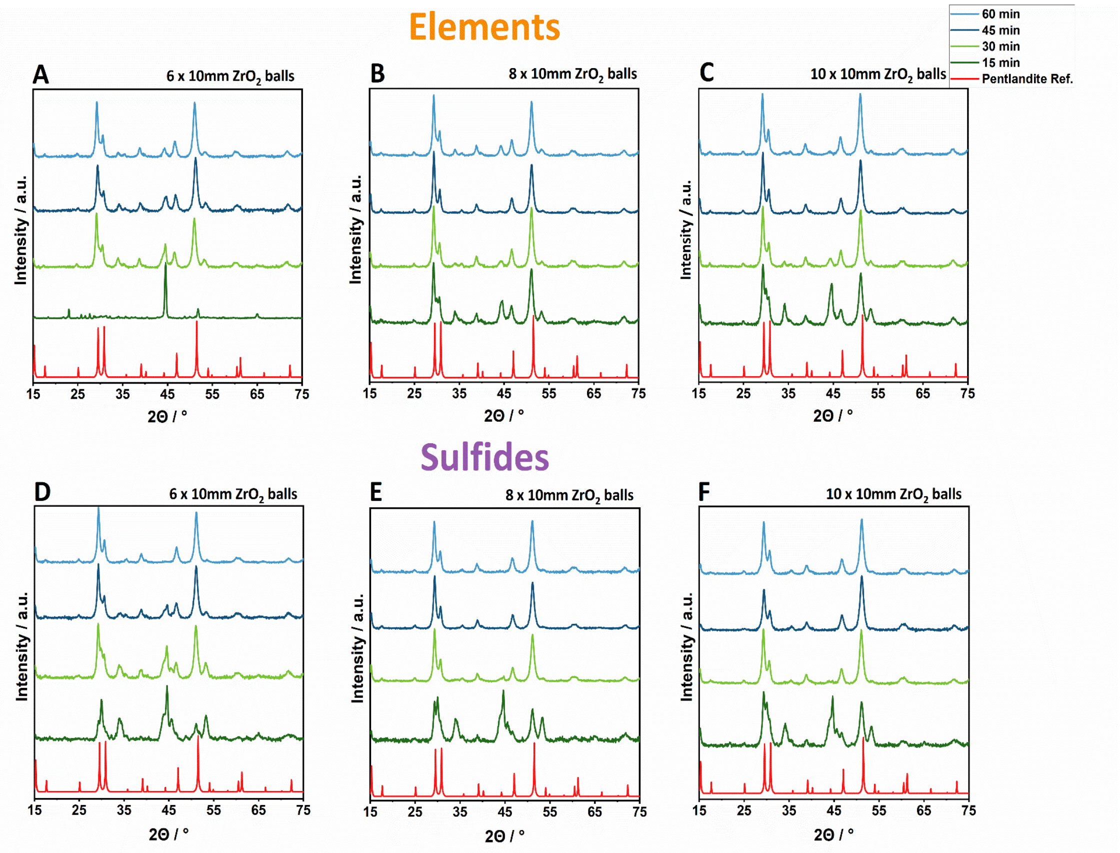

Fig. S1: Powder X-ray diffractogram measurements of Pn synthesized at the different timescales with (A) six, (B) eight and (C) ten 10 mm ZrO_2 balls added to an elemental reaction mixture; (D) six, (E) eight, (F) ten 10 mm ZrO_2 balls added to a sulfidic reaction mixture. [1]

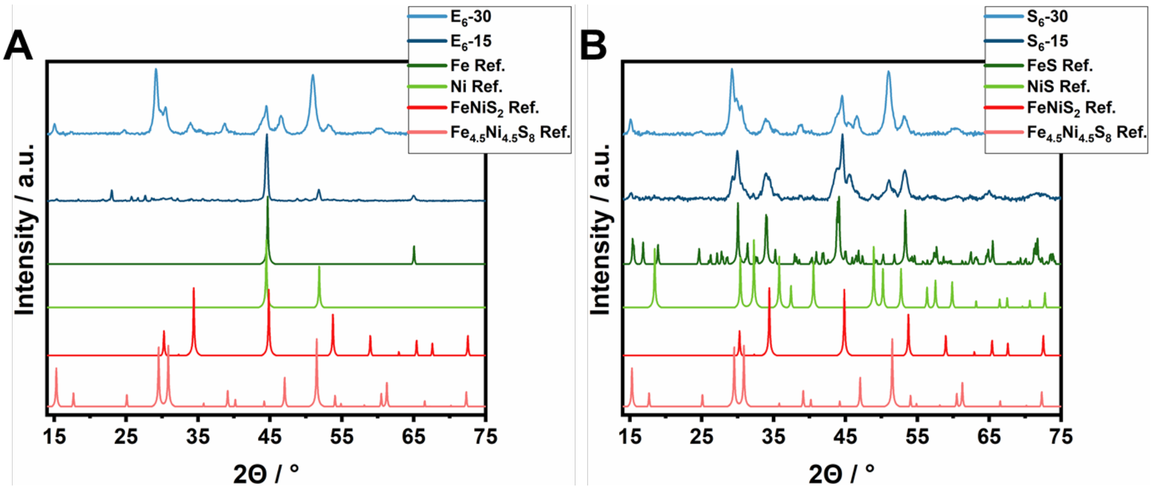

Fig. S2: Phase analysis of the synthesized pentlandite materials using a) an elemental reaction mixtures. b) a sulfidic reaction mixture. Powder diffractograms are compared to the reference powder diffractograms of $Fe_{4.5}Ni_{4.5}S_8$[1], $FeNiS_2$[2], Fe[3], Ni[4], NiS (Millerite)[5] and FeS (Pyrrhotite)[6].

Rietveld analysis of the synthesized Pn materials

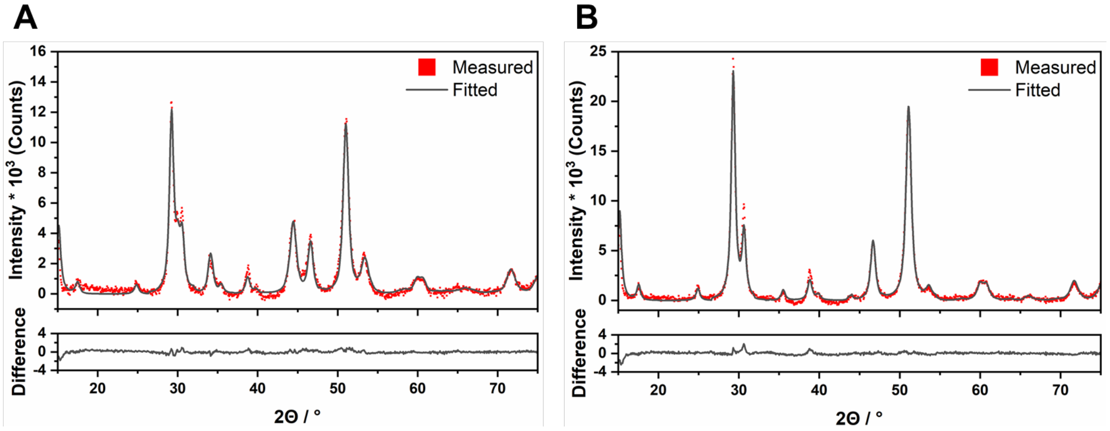

Fig. S3: Examples of Rietveld analysis data performed at the samples A) E_8-15 and B) E_8-45.

Table S1: Pentlandite yields of the synthesized materials using an elemental reaction mixture (E_x-Y) determined via Rietveld analysis. The remaining phases can be ascribed to sulfidic impurities like $FeNiS_2$.

Sample	Pentlandite yield [%]
E_6-15	-
E_6-30	62,1
E_6-45	74,8
E_6-60	87,5
E_8-15	67,0
E_8-30	86,1
E_8-45	99,8
E_8-60	86,5
E_{10}-15	56,0
E_{10}-30	85,7
E_{10}-45	99,6
E_{10}-60	99,3

Table S2: Pentlandite yields of the synthesized materials using a sulfidic reaction mixture (S_x-Y) determined via Rietveld analysis. The remaining phases can be ascribed to sulfidic impurities like $FeNiS_2$.

Sample	Pentlandite yield [%]
S_6-15	18,3
S_6-30	50,7
S_6-45	72,2
S_6-60	99,3
S_8-15	33,1
S_8-30	90,7
S_8-45	100
S_8-60	99,3
S_{10}-15	34,4
S_{10}-30	99,7
S_{10}-45	99,5
S_{10}-60	99,7

Differential Scanning Calorimetry of the synthesized Pn materials

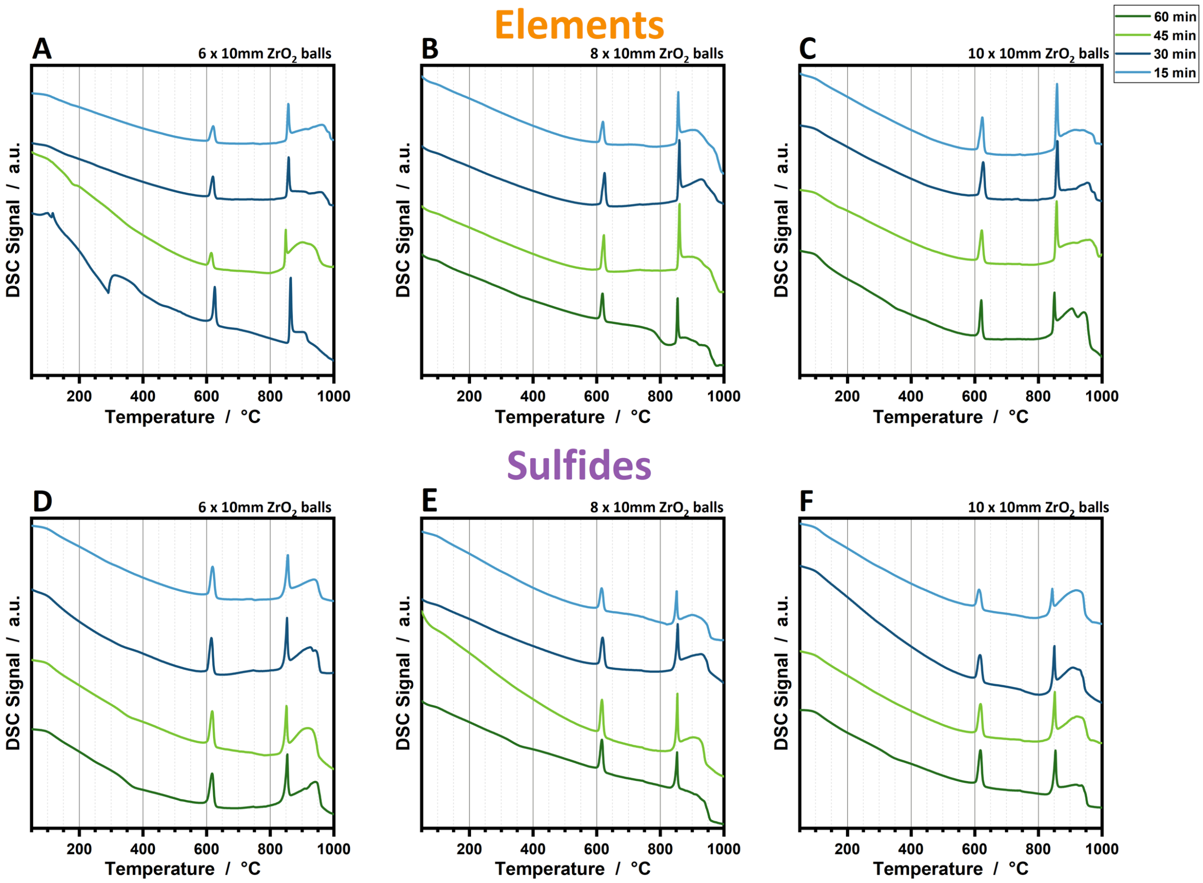

Fig. S4: Differential scanning calometry measurements of Pn synthesized across the different timescales with a) six 10 mm ZrO_2 balls added to an elemental reaction mixture. (b) eight 10 mm ZrO_2 balls added to an elemental reaction mixture. (c) ten 10 mm ZrO_2 balls added to an elemental reaction mixture. (d) six 10 mm ZrO_2 balls added to a sulfidic reaction mixture. (e) eight 10 mm ZrO_2 balls added to a sulfidic reaction mixture. (f) ten 10 mm ZrO_2 balls added to a sulfidic reaction mixture.

Phase transitions of the synthesized Pn materials from the elemental reactants according to Differential Scanning Calorimetry

Table S3: Phase transition onset temperatures of the synthesized E_x-Y Pn materials.

**Sample**	**T_1 low→high (°C)**	**T_2 breakdown (°C)**
**E_6-15**	617	858
**E_6-30**	603	845
**E_6-45**	607	851
**E_6-60**	606	851
**E_8-15**	609	850
**E_8-30**	612	855
**E_8-45**	613	854
**E_8-60**	608	852
**E_{10}-15**	612	845
**E_{10}-30**	610	851
**E_{10}-45**	614	853
**E_{10}-60**	611	852
**Ref.[7]**	617	859

Phase transitions of the synthesized Pn materials from the sulfidic reactants according to Differential Scanning Calorimetry

Table S4: Phase transition onset temperatures of the synthesized S_x-Y Pn materials.

Sample	T_1 low→high (°C)	T_2 breakdown (°C)
**S_6-15**	603	846
**S_6-30**	605	843
**S_6-45**	603	845
**S_6-60**	606	845
**S_8-15**	606	846
**S_8-30**	607	848
**S_8-45**	608	847
**S_8-60**	605	844
**S_{10}-15**	606	845
**S_{10}-30**	606	844
**S_{10}-45**	603	842
**S_{10}-60**	602	837
**Ref.[7]**	617	859

Differential Scanning Calorimetry analysis of an unreacted elemental reaction mixture

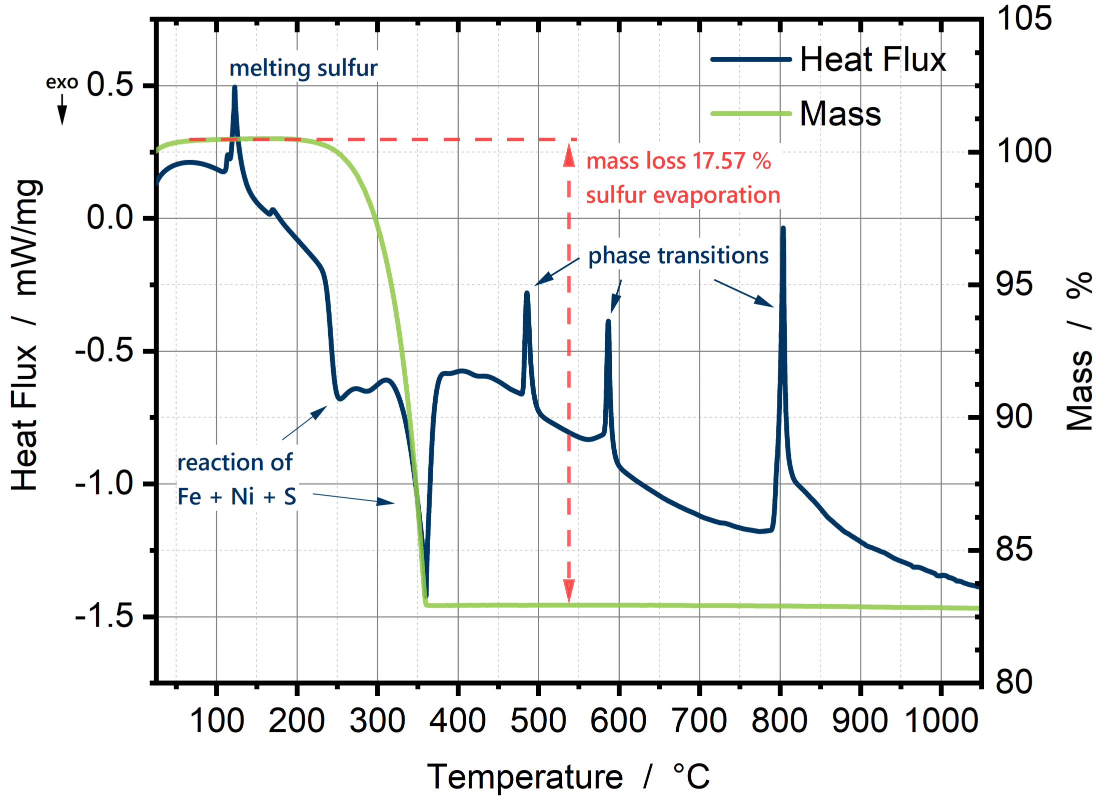

Fig. S5: DSC measurement of the unreacted elemental reaction mixture.

Scanning Electron Microscopy of the synthesized Pn materials from an elemental reaction mixture

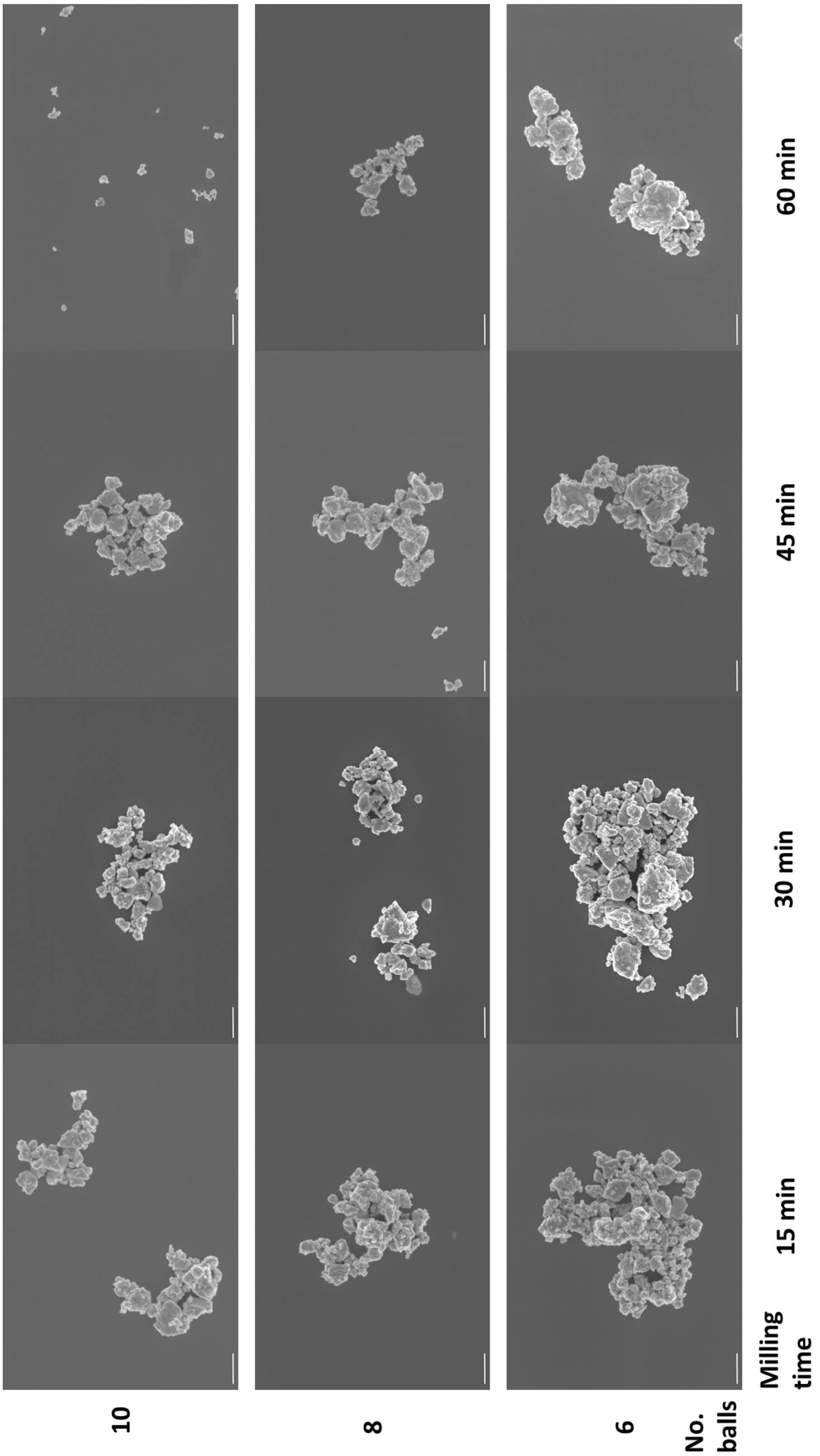

Fig. S6: SEM images of the synthesized Pn materials synthesized from the elemental reaction mixture. The scale bar is 1 µm.

Scanning Electron Microscopy of the synthesized Pn materials from a sulfidic reaction mixture

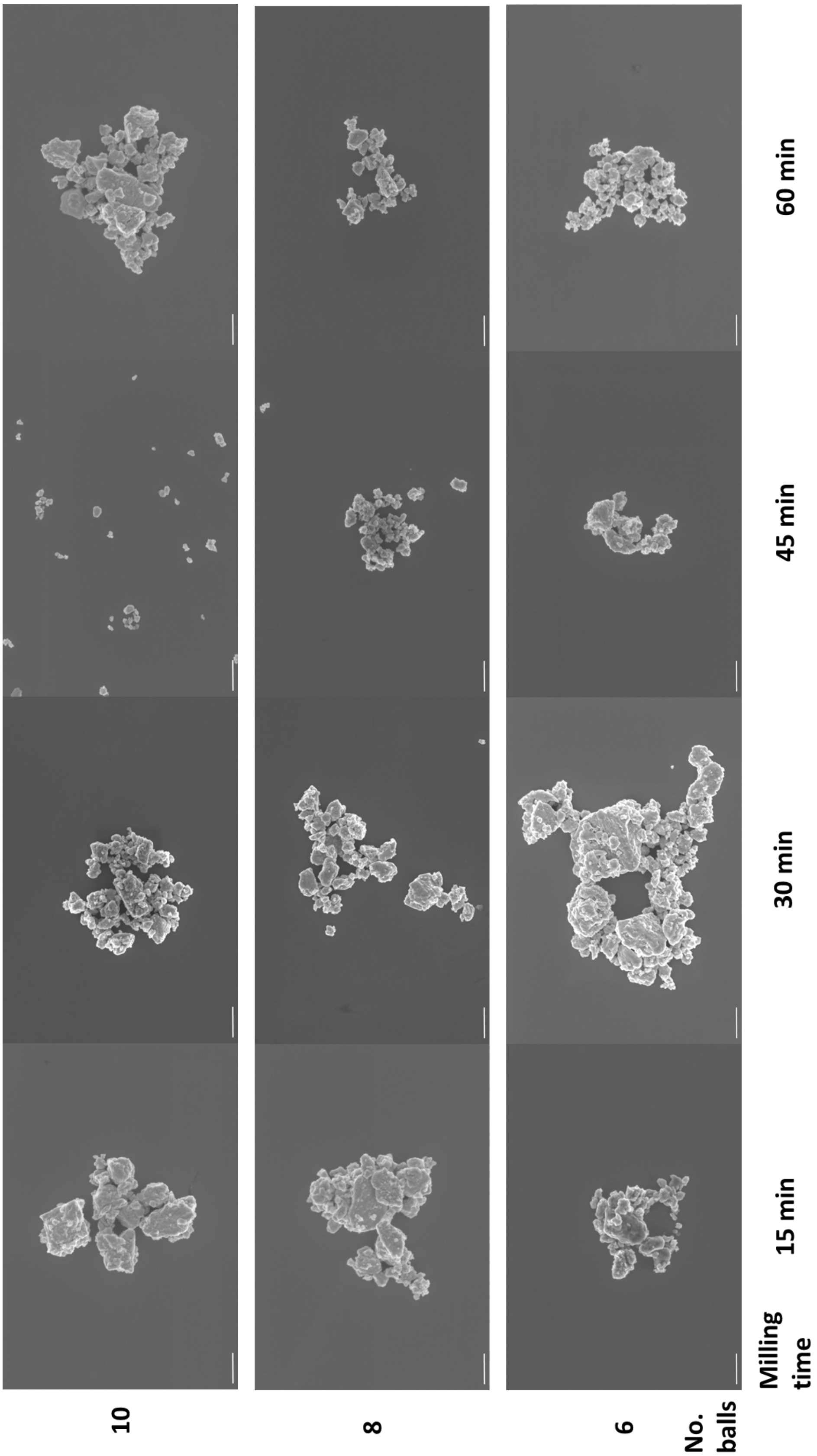

Fig. S7: SEM images of the synthesized Pn materials synthesized from the sulfidic reaction mixture. The scale bar is 1 µm.

Disc centrifuge measurements of the synthesized materials

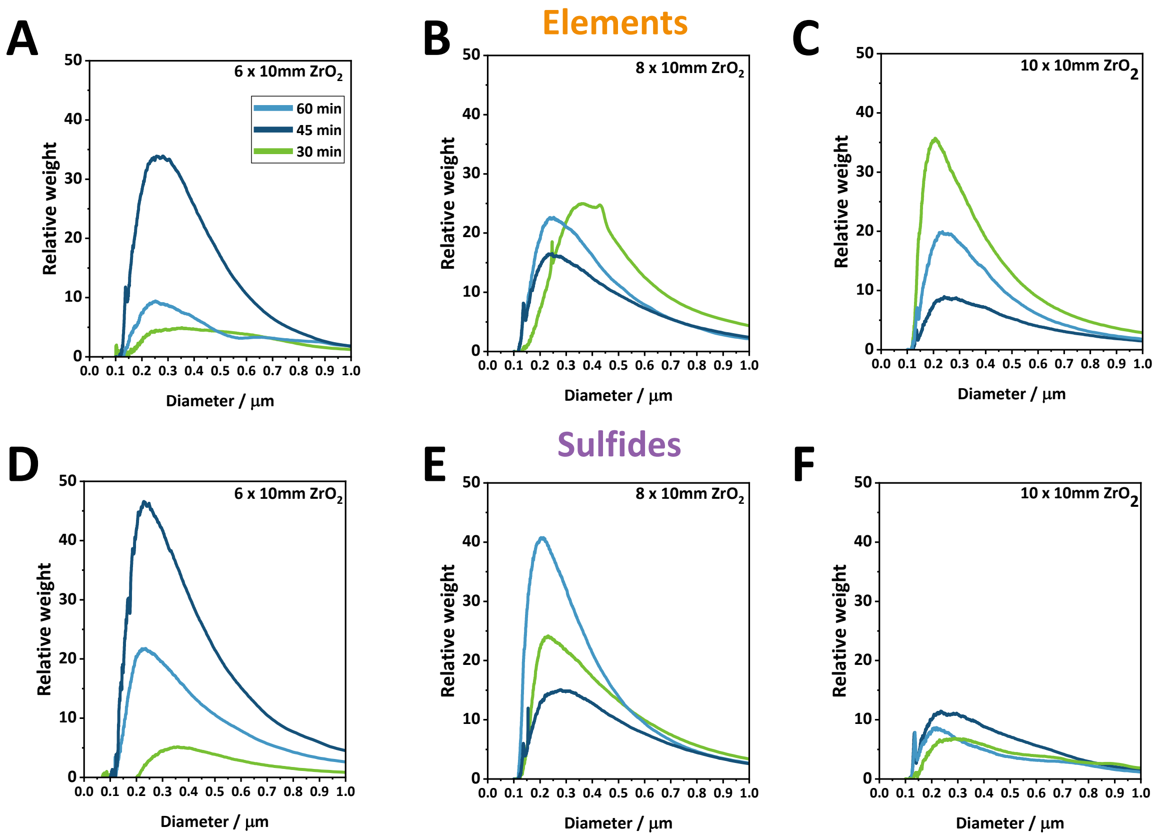

Fig. S8: Disc centrifuge measurements of Pn synthesized across different timescales employing (A) six 10 mm ZrO_2 balls added to an elemental mixture. (B) eight 10 mm ZrO_2 balls added to an elemental mixture. (C) ten 10 mm ZrO_2 balls added to an elemental mixture. (D) six 10 mm ZrO_2 balls added to a sulfide mixture. (E) eight 10 mm ZrO_2 balls added to a sulfide mixture. (F) ten 10 mm ZrO_2 balls added to a sulfide mixture. Since samples prepared by 15 min of milling do not show a pure pentlandite phase, discussion of their particle distribution is omitted.

Particle size analysis of the synthesized Pn materials prepared through an elemental reaction mixture

Table S5: Mean particles sizes of the synthesized pentlandite materials from the elemental reaction mixture determined via disc centrifuge.

Sample	Particle size (nm)
E_6-30	358 ± 30
E_6-45	232 ± 20
E_6-60	229 ± 20
E_8-30	363 ± 25
E_8-45	244 ± 30
E_8-60	240 ± 30
E_{10}-30	208 ± 15
E_{10}-45	243 ± 30
E_{10}-60	235 ± 30

Particle size analysis of the synthesized Pn materials prepared through a sulfidic reaction mixture

Table S6: Mean particles sizes of the synthesized pentlandite materials from the sulfidic reaction mixture determined via disc centrifuge.

Sample	Particle size (nm)
**S_6-30**	350 ± 60
**S_6-45**	270 ± 25
**S_6-60**	253 ± 25
**S_8-30**	229 ± 20
**S_8-45**	251 ± 25
**S_8-60**	209 ± 15
**S_{10}-30**	294 ± 50
**S_{10}-45**	237 ± 40
**S_{10}-60**	218 ± 20

Non-noble metal HER electrocatalysts in a PEM electrolyzer reports in literature

Table S7: Comparison of the performance of non-noble metal HER electrocatalysts in a PEM electrolyzer reports in literature.

Catalyst	Loading / mg cm^{-2}	j / A cm^{-2}	Cell potential / V	Membrane	Anodic catalyst (Loading / mg cm^{-2})	Temperature / °C	Citation
**E_8-45**	1.0	1.0	1.95	Nafion HP	Ir-black (2.0)	80	This work
**E_8-45**	2.0	1.0	1.91	Nafion HP	Ir-black (2.5)	80	This work
**E_8-45**	4.0	1.0	1.93	Nafion HP	Ir-black (2.5)	80	This work
**47 wt.% MoS_2/Vulcan**	5.0	0.3	2.00	Nafion 117	IrO_2 (2.0)	80	[8]
**Pyrite/C**	4.0	1.0	2.10	Nafion 115	IrO_2 (2.0)	80	[9]
**Greigite/C**	4.0	1.0	2.13	Nafion 115	IrO_2 (2.0)	80	[9]
**Pyrrhotite/C**	4.0	1.0	2.16	Nafion 115	IrO_2 (2.0)	80	[9]
**RuS_2@MoS_2**	2.0	1.0	1.65	Nafion 112	IrO_2 (2.0)	80	[10]
**MoP|S**	3.0	1.1	2.00	Nafion 115	IrO_2 (2.0)	80	[11]
**Mo_3S_{13}-NCNT**	3.0	1.4	2.00	Nafion 212	IrO_2 (1.5)	80	[12]

Zr quantification through PIXE

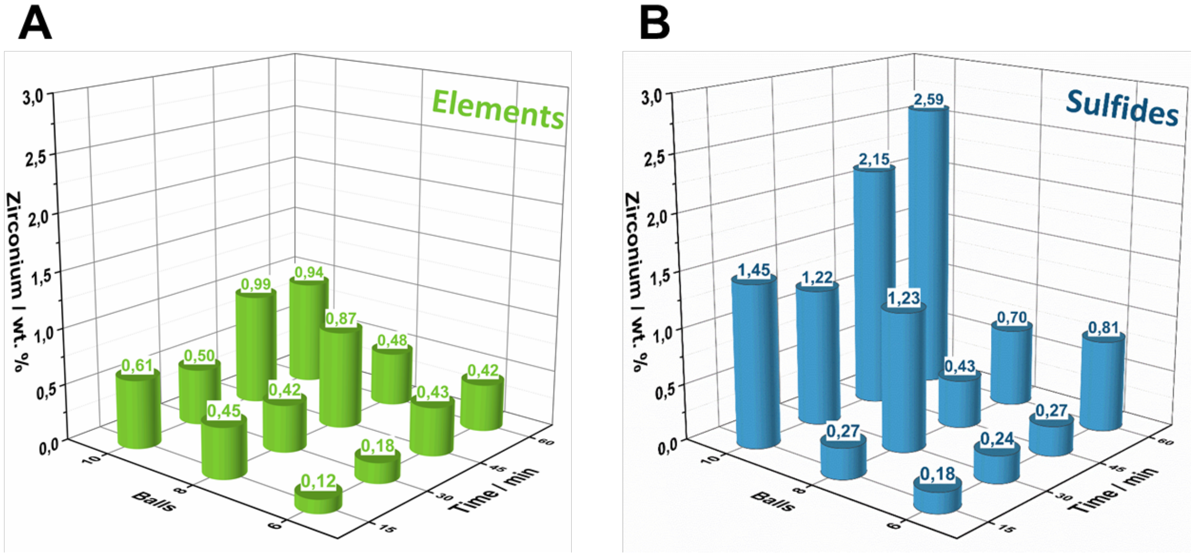

Fig. S9: Quantification of Zr abrasion impurities via PIXE of the synthesized Pn samples synthesized by employing a) elemental reaction mixtures. b) sulfidic reaction mixtures.

EDX mapping analysis of the synthesized Pn materials prepared through an elemental reaction mixture using six ZrO_2 milling balls

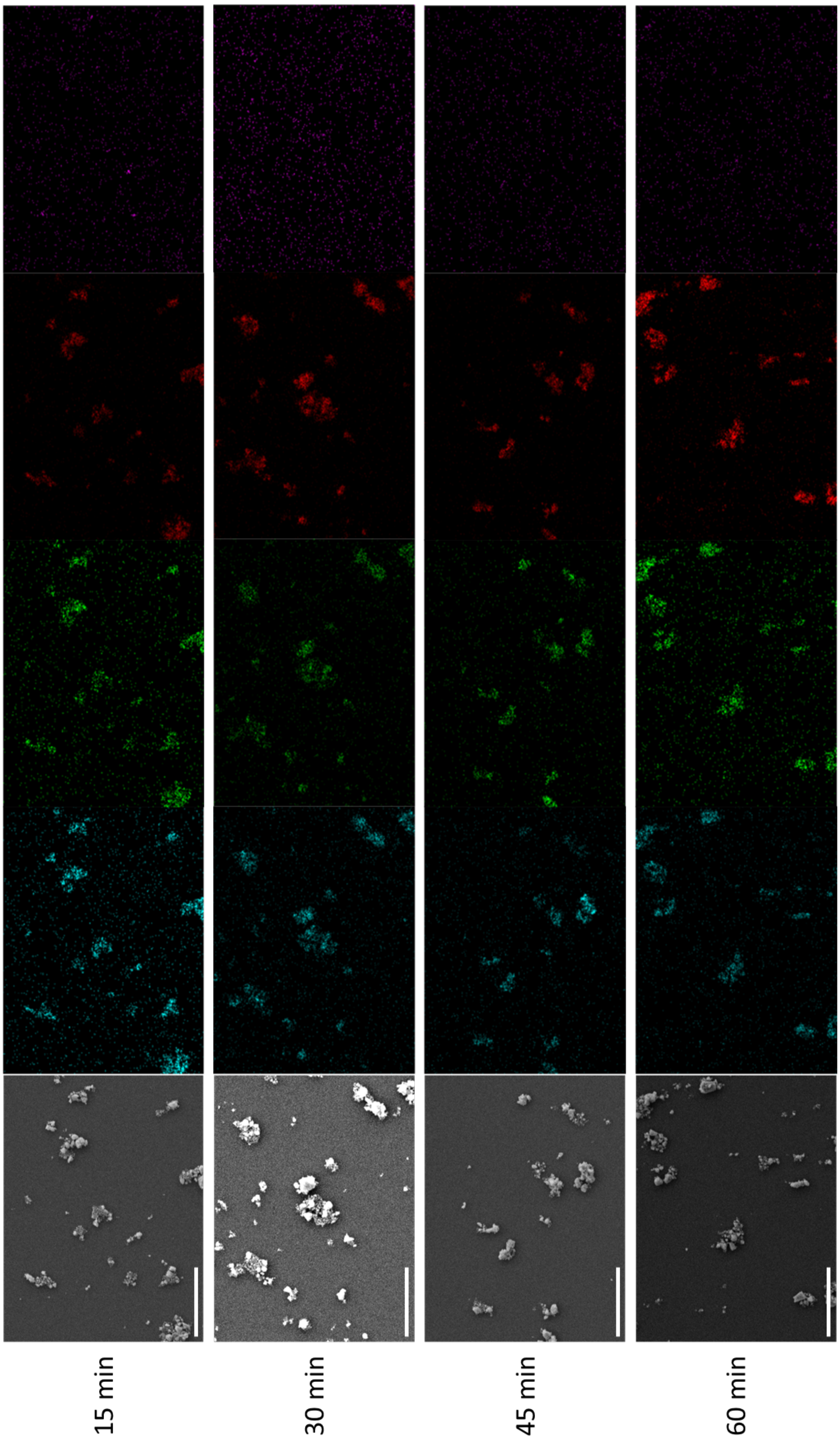

Fig. S10: EDX elemental mapping of the synthesized Pn materials synthesized from the elemental reaction mixture using six milling balls. The scale bar is 25 µm. EDX mappings reveal the presence of the elements Fe (blue), Ni (green), sulfur (red) and zirconium (violet).

EDX mapping analysis of the synthesized Pn materials prepared through an elemental reaction mixture using eight ZrO_2 milling balls

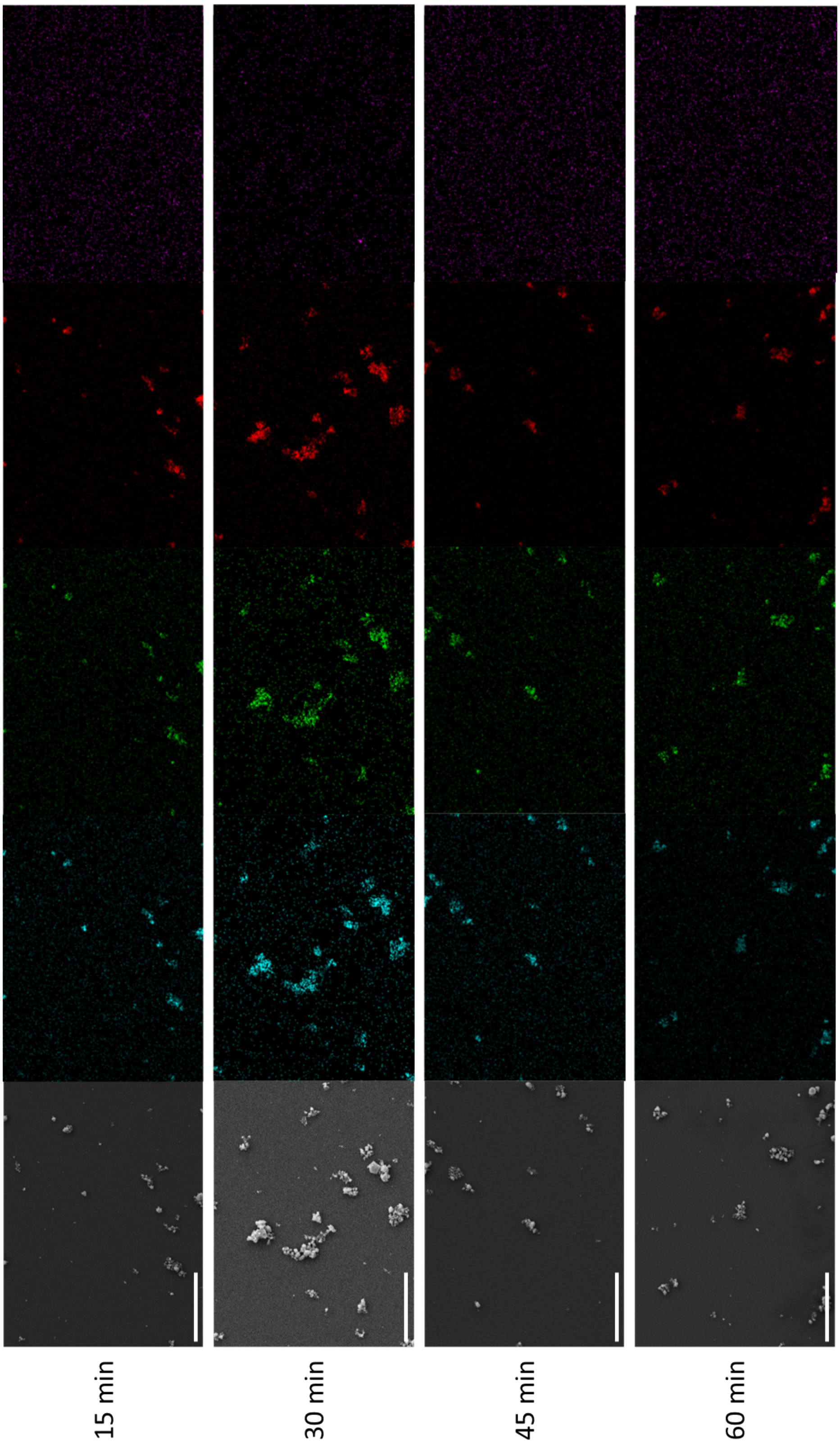

Fig. S11: EDX elemental mapping of the synthesized Pn materials synthesized from the elemental reaction mixture using eight milling balls. The scale bar is 25 µm. EDX mappings reveal the presence of the elements Fe (blue), Ni (green), sulfur (red) and zirconium (violet).

EDX mapping analysis of the synthesized Pn materials prepared through an elemental reaction mixture using ten ZrO_2 milling balls

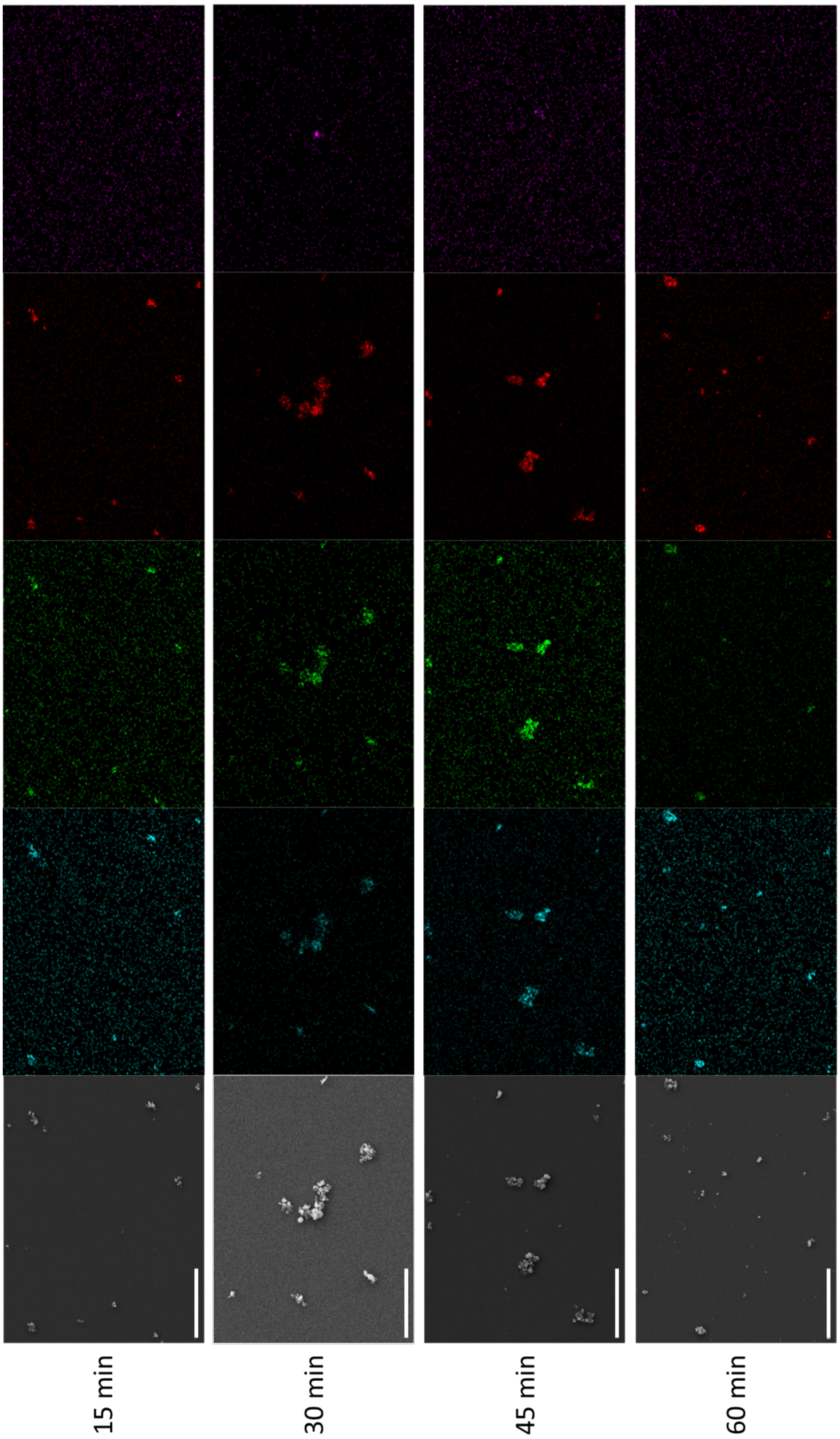

Fig. S12: EDX elemental mapping of the synthesized Pn materials synthesized from the elemental reaction mixture using ten milling balls. The scale bar is 25 µm. EDX mappings reveal the presence of the elements Fe (blue), Ni (green), sulfur (red) and zirconium (violet).

EDX mapping analysis of the synthesized Pn materials prepared through a sulfidic reaction mixture using six ZrO_2 milling balls

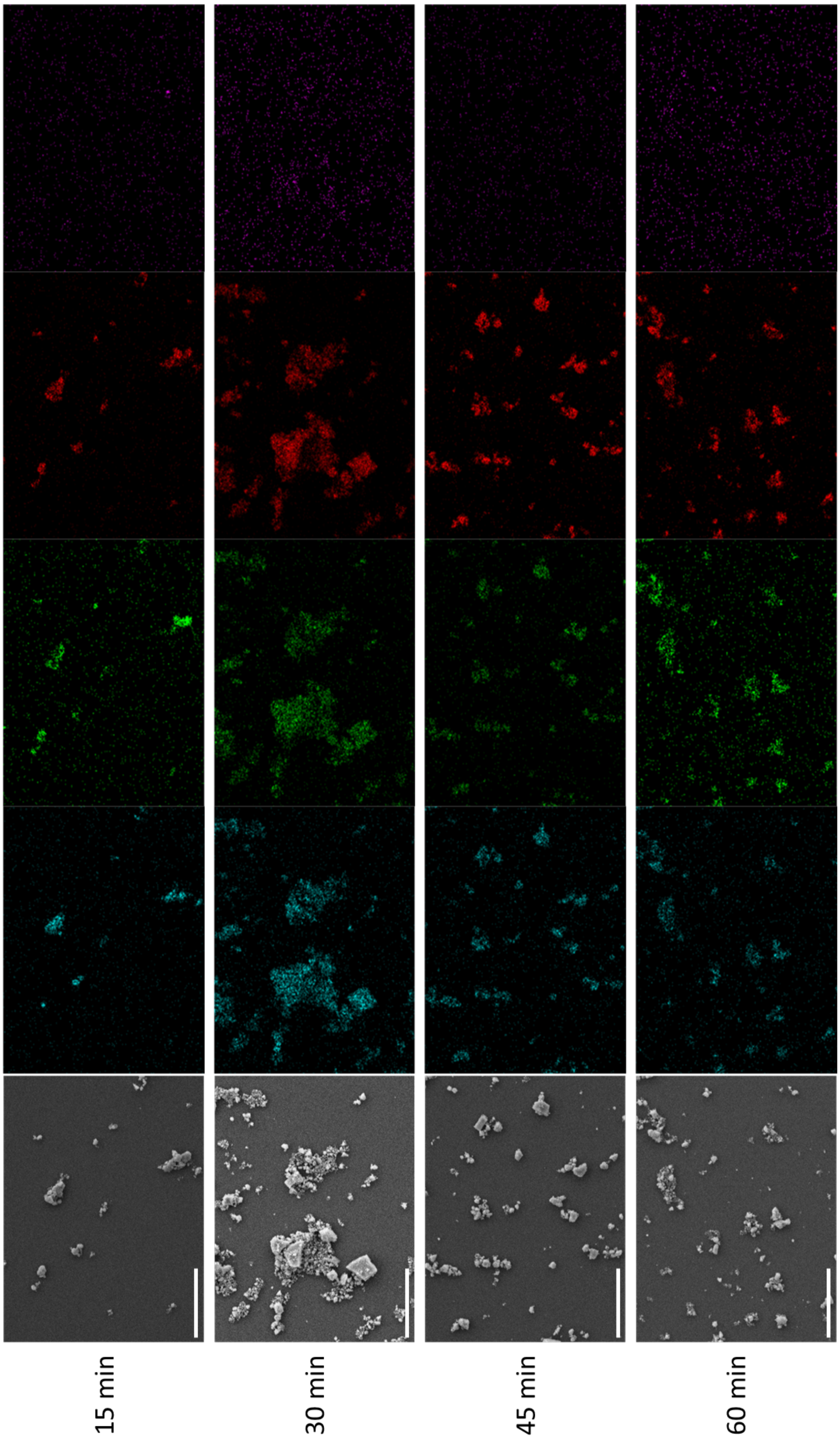

Fig. S13: EDX elemental mapping of the synthesized Pn materials synthesized from the sulfidic reaction mixture using six milling balls. The scale bar is 25 µm. EDX mappings reveal the presence of the elements Fe (blue), Ni (green), sulfur (red) and zirconium (violet).

EDX mapping analysis of the synthesized Pn materials prepared through a sulfidic reaction mixture using eight ZrO_2 milling balls

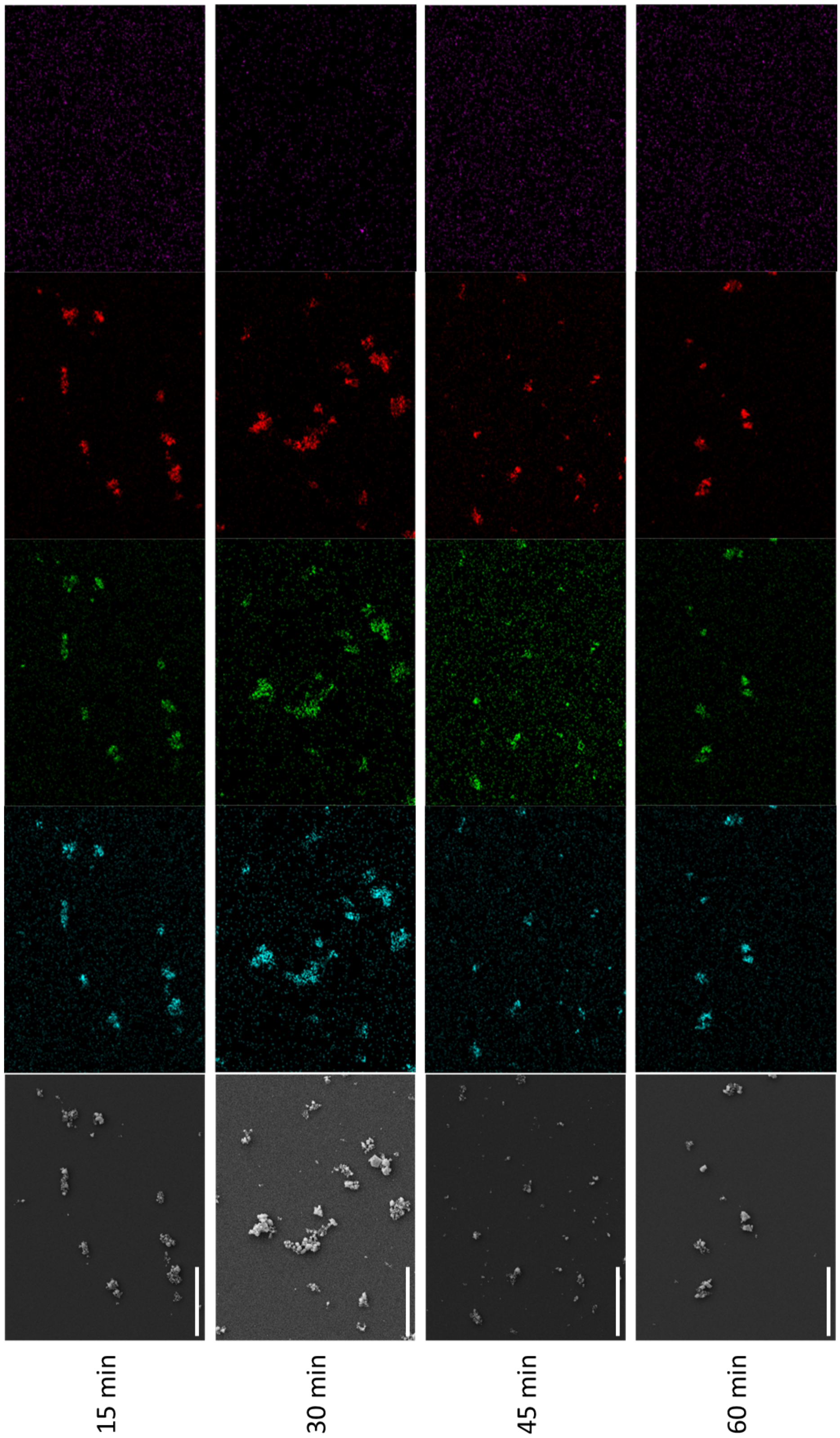

Fig. S14: EDX elemental mappng of the synthesized Pn materials synthesized from the sulfidic reaction mixture using eight milling balls. The scale bar is 25 µm. EDX mappings reveal the presence of the elements Fe (blue), Ni (green), sulfur (red) and zirconium (violet).

EDX mapping of the synthesized Pn materials prepared through a sulfidic reaction mixture using ten ZrO_2 milling balls

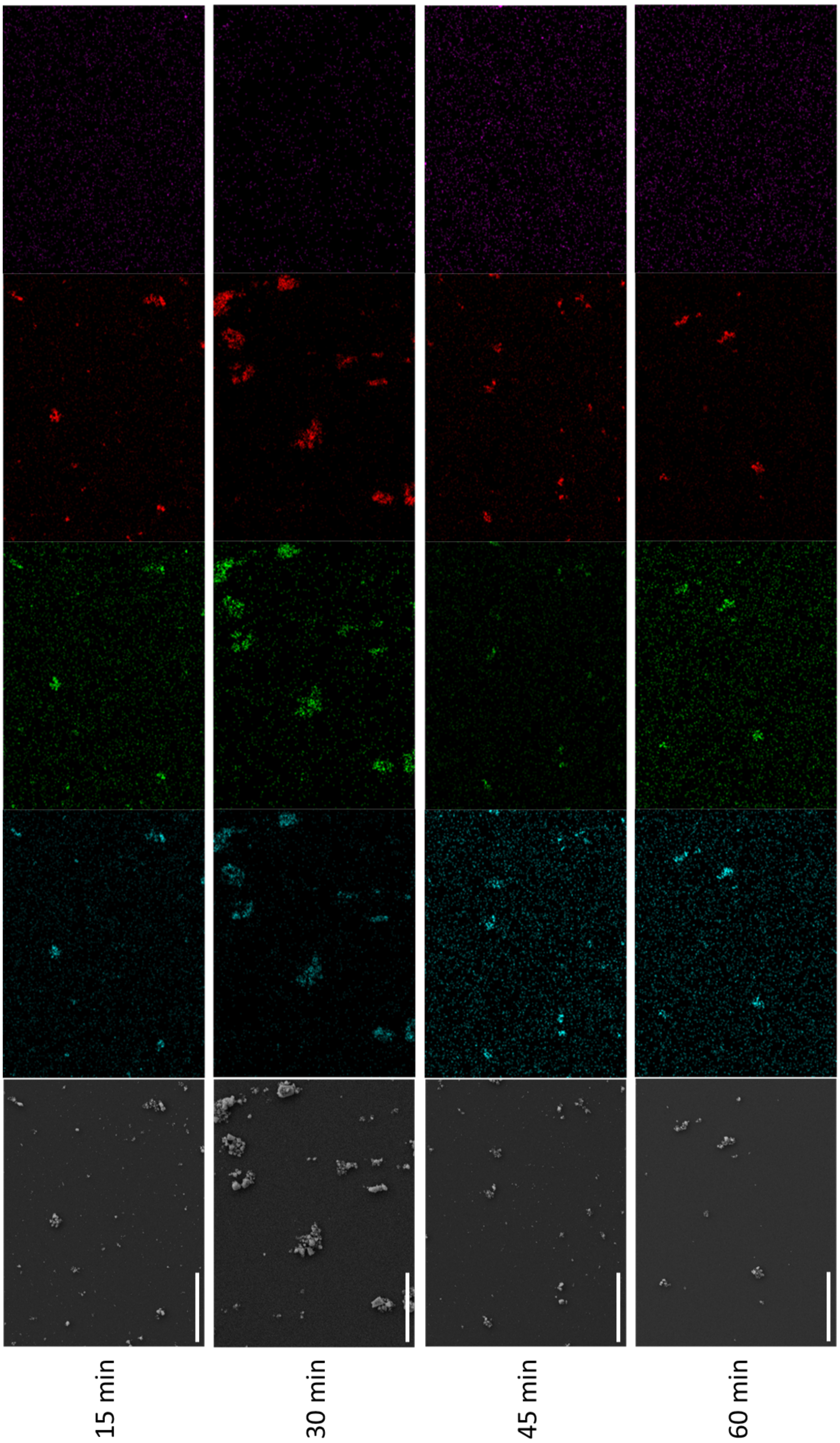

Fig. S15: EDX elemental mapping of the synthesized Pn materials synthesized from the sulfidic reaction mixture using ten milling balls. The scale bar is 25 µm. EDX mappings reveal the presence of the elements Fe (blue), Ni (green), sulfur (red) and zirconium (violet).

Gas and Temperature monitoring in the milling vessel starting from elemental and sulfidic reactant mixtures

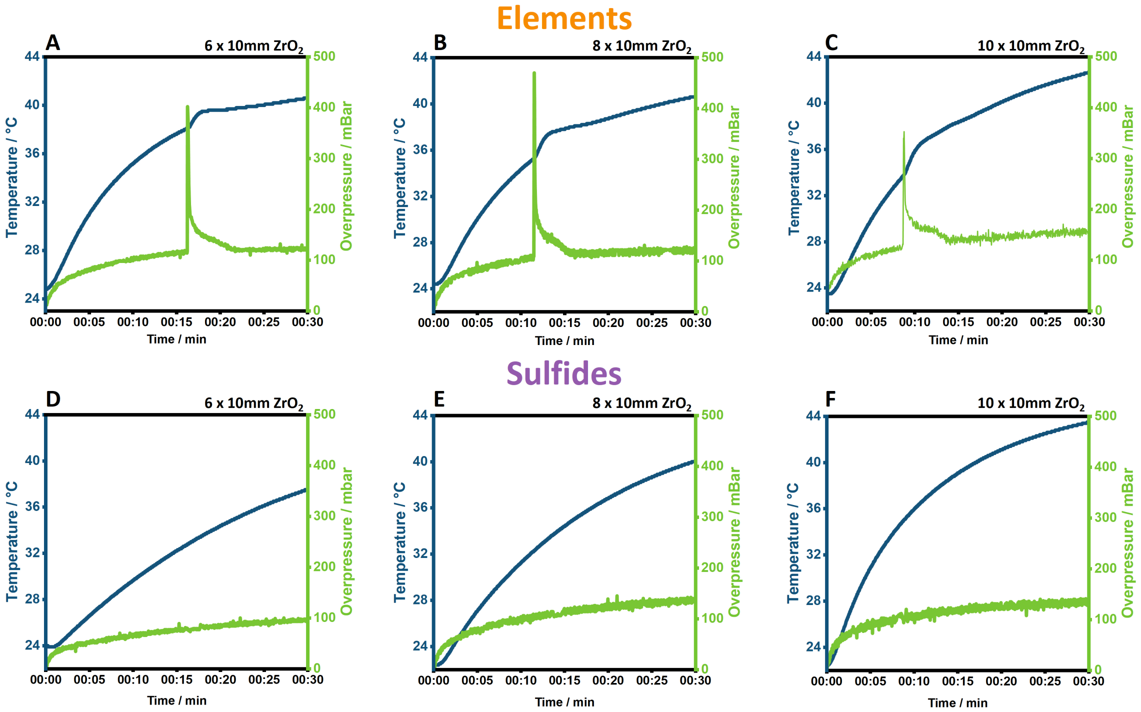

Fig. S16: Pressure- and temperature measurements during Pn synthesis employing (A) six 10 mm ZrO_2 balls added to an elemental reaction mixture. (B) eight 10 mm ZrO_2 balls added to an elemental reaction mixture. (C) ten 10 mm ZrO_2 balls added to an elemental reaction mixture. (D) six 10 mm ZrO_2 balls added to a sulfidic reaction mixture. (F) eight 10 mm ZrO_2 balls added to a sulfidic reaction mixture. (F) ten 10 mm ZrO_2 balls added to a sulfidic reaction mixture.

PXRDs of the synthesized Pn materials generated through the Gas and Temperature monitoring experiments

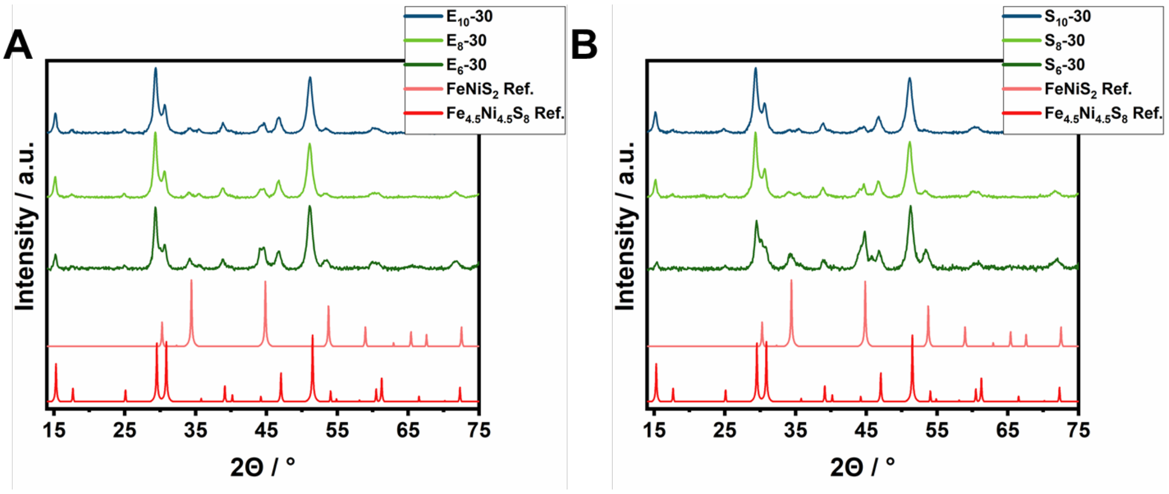

Fig. S17: Powder XRD analysis of the synthesized Pn materials from the pressure- and temperature measurements compared to the reference powder pattern of $Fe_{4.5}Ni_{4.5}S_8$[1] and $FeNiS_2$[2].

Raman investigation of the S_8-Y samples

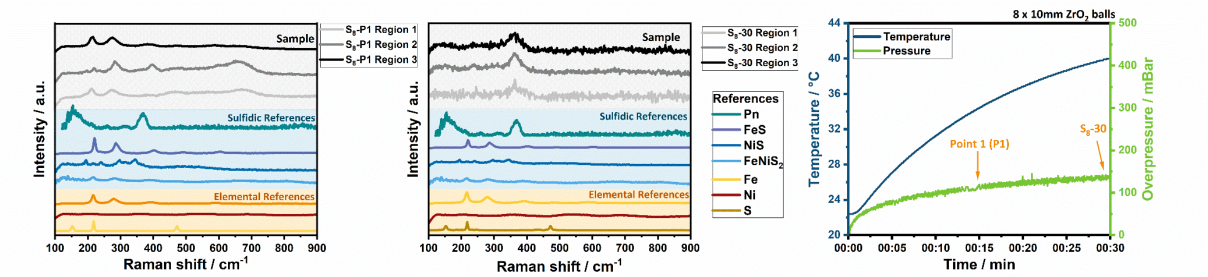

Fig. S18: Raman spectra of the obtained S_8-Y samples at three different time points of the mechanochemical reaction: at the half-point of the mechanochemical reaction (A) and after 30 min of milling (B). The respective points are also shown for clarity in (C).

Electrochemical cell for PEM investigation

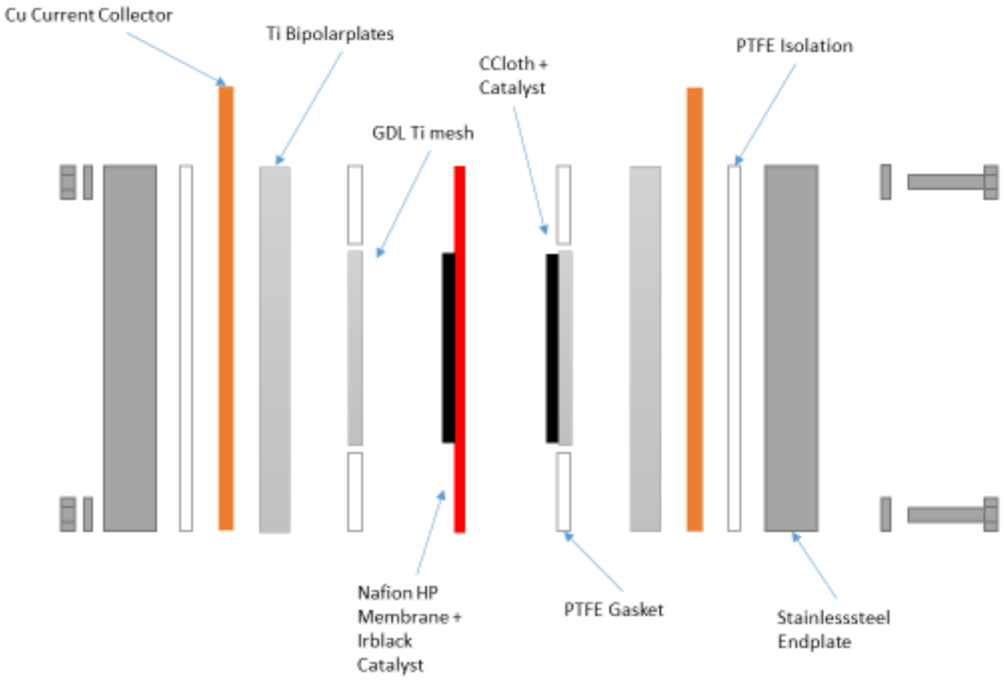

Fig. S19: Schematic presentation of the used PEM electrolyzer.

Electrochemical HER using the synthesized materials in a PEM electrolyzer

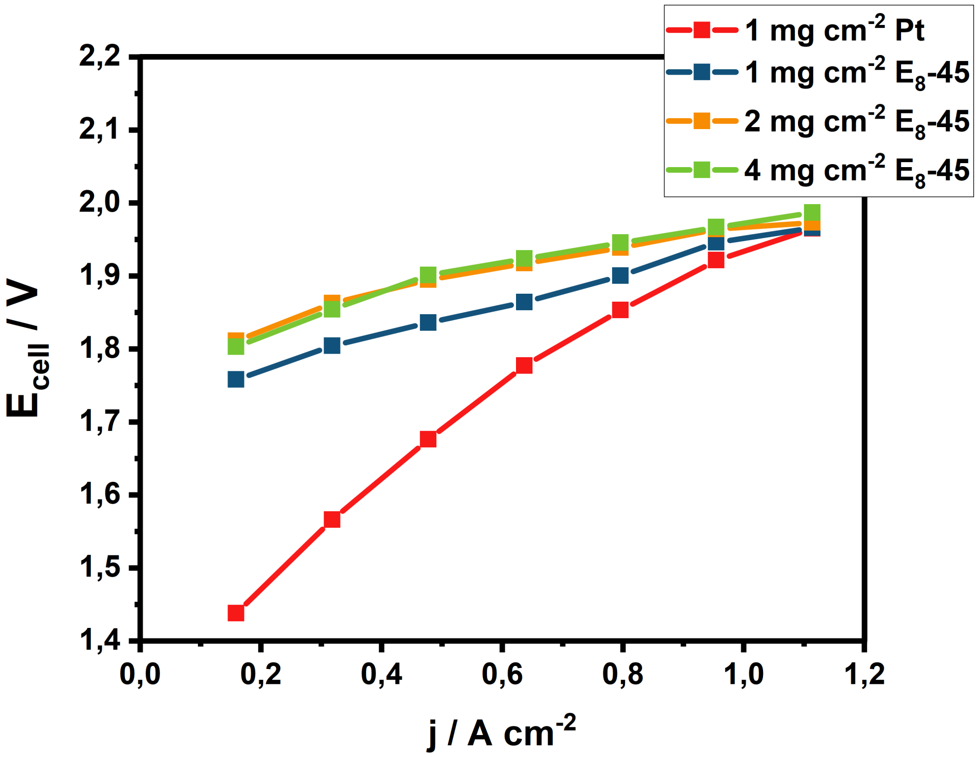

Fig. S20: : Polarization curves of Pt/C (1 mg cm^{-2} loading) and E_8-45 with different loadings from 1-4 mg cm^{-2} recorded at a temperature of 80 °C (A).

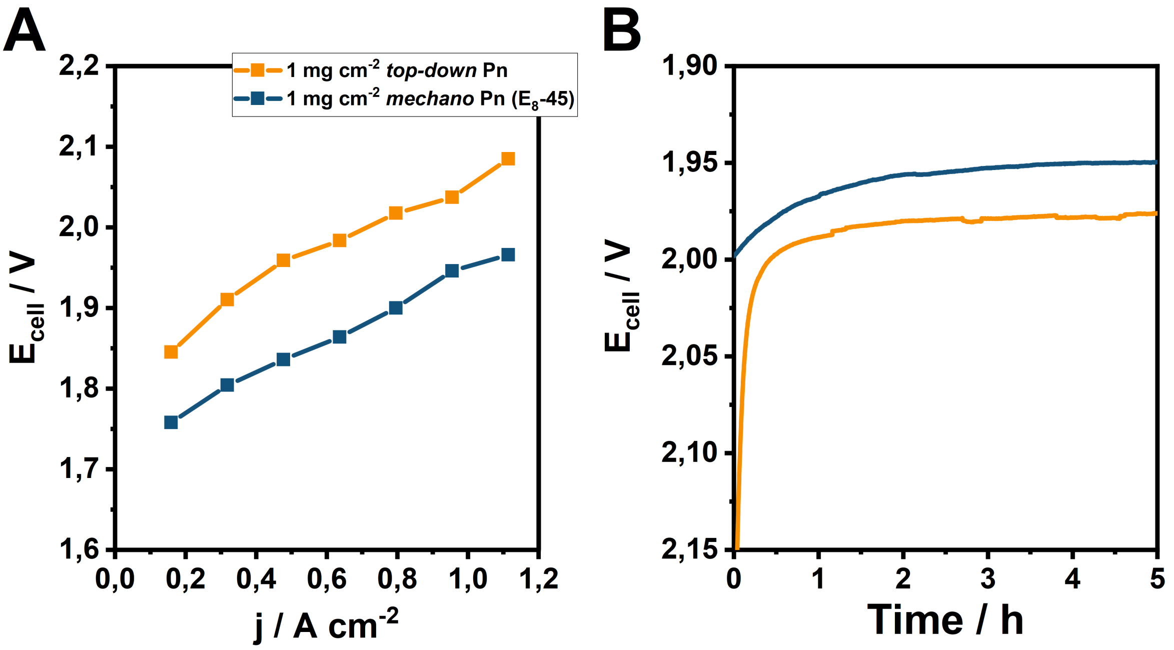

Fig. S21: Polarization curves of top-down synthesized Pn and mechano Pn (E_8-45) at a catalytic loading of 1 mg cm^{-2} recorded at a temperature of 80 °C (A). Chronopotentiometry of top-down synthesized Pn and mechano-Pn (E_8-45) at a catalytic loading of 1 mg cm^{-2} at 80 °C for 5 h at an applied current of 1 A cm^{-2} (B).

References

1 A. D. Pearson and M. J. Buerger, *Am. Mineral.*, 1956, **41**, 804.
2 N. Alsen, *Geologiska Foereningens i Stockholm Foerhandlingar*, 1925, **47**, 19.
3 D. R. Wilburn and W. A. Bassett, *Am. Mineral.*, 1978, **63**, 591.
4 J. Zemann, *Acta Cryst*, 1965, **18**, 139.
5 J. D. Grice and R. B. Ferguson, *Canad. Mineral.*, 1974, **12**, 248â€“252.
6 Masayasu Tokonami, Katsuhisa Nishiguchi and Nobuo Morimoto, *Am. Mineral.*, 1972, **57**, 1066.
7 A. Sugaki and A. Kitakaze, *Am. Mineral.*, 1998, **83**, 133.
8 T. Corrales-Sánchez, *J. Hydrog. Energ.*, 2014, **39**, 20837.
9 C. Di Giovanni, Á. Reyes-Carmona, A. Coursier, S. Nowak, J.–M. Grenèche, H. Lecoq, L. Mouton, J. Rozière, D. Jones, J. Peron, M. Giraud, C. Tard, *ACS Catal.*, 2016, **6**, 2626.
10 M. Sarno and E. Ponticorvo, *Int. J. Hydrog. Energ.*, 2019, **44**, 4398.
11 J. W. D. Ng, T. R. Hellstern, J. Kibsgaard, A. C. Hinckley, J. D. Benck and T. F. Jaramillo, *ChemSusChem*, 2015, **8**, 3512.
12 P. K. R. Holzapfel, M. Bühler, D. Escalera-López, M. Bierling, F. D. Speck, K. J. J. Mayrhofer, S. Cherevko, C. V. Pham and S. Thiele, *Small*, 2020, **16**, e2003161.
